# The surface modification of the silica-coated magnetic nanoparticles and their application in molecular diagnostics of virus infection

**DOI:** 10.1038/s41598-024-64839-2

**Published:** 2024-06-23

**Authors:** A. Zeleňáková, V. Zeleňák, E. Beňová, B. Kočíková, N. Király, P. Hrubovčák, J. Szűcsová, Ľ. Nagy, M. Klementová, J. Mačák, V. Závišová, J. Bednarčík, J. Kupčík, A. Jacková, D. Volavka, J. Košuth, Š. Vilček

**Affiliations:** 1 Institute of Physics, Faculty of Science, P.J. Šafárik University, Park Angelinum 9, 04001 Košice, Slovakia; 2 Institute of Chemistry, Faculty of Science, P.J. Šafárik University, Moyzesova 11, 04001 Košice, Slovakia; 3 Department of Epizootiology, Parasitology and Public Health Protection, University of Veterinary Medicine and Pharmacy in Košice, Komenského 73, 04181 Košice, Slovakia; 4 Institute of Physics of the CAS, v.v.i., Na Slovance 1999/2, 182 21 Praha 8, Czech Republic; 5 Synlab Slovakia s. r. o Department of Clinical Microbiology, Opatovská Cesta 10, 04001 Košice, Slovakia; 6 Institute of Experimental Physics, Slovak Academy of Sciences, Watsonova 47, 04001 Košice, Slovakia; 7 Institute of Biology and Ecology, Faculty of Science, P.J. Šafárik University, Šrobárova 2, 04154 Košice, Slovakia

**Keywords:** Materials science, Health care

## Abstract

The study presents a series of examples of magnetic nanoparticle systems designed for the diagnosis of viral diseases. In this interdisciplinary work, we describe one of the most comprehensive synthetic approaches for the preparation and functionalization of smart nanoparticle systems for rapid and effective RT-PCR diagnostics and isolation of viral RNA. Twelve different organic ligands and inorganic porous silica were used for surface functionalization of the Fe_3_O_4_ magnetic core to increase the number of active centres for efficient RNA binding from human swab samples. Different nanoparticle systems with common beads were characterized by HRTEM, SEM, FT-IR, XRD, XPS and magnetic measurements. We demonstrate the application of the fundamental models modified to fit the experimental zero-field cooling magnetization data. We discuss the influence of the nanoparticle shell parameters (morphology, thickness, ligands) on the overall magnetic performance of the systems. The prepared nanoparticles were tested for the isolation of viral RNA from tissue samples infected with hepatitis E virus—HEV and from biofluid samples of SARS-CoV-2 positive patients. The efficiency of RNA isolation was quantified by RT-qPCR method.

## Introduction

The development of materials containing magnetic nanoparticles (MNPs) has focused intense research efforts due to their applications in catalysis^1,2^, magnetic separation^3^, and especially in biomedicine: as enhancing contrast agents for magnetic resonance imaging (MRI)^4,5^, targeted drug delivery^6,7^, the treatment of cancer by localized therapeutic hyperthermia^8,9^ etc. For these applications, the magnetic iron oxide particles consisting of magnetite (Fe_3_O_4_) or maghemite (γ-Fe_2_O_3_) and appropriately coated should exhibit superparamagnetic behavior, significant saturation magnetization, high magnetic susceptibility and biocompatibility^10,11^. Magnetic properties allow particles movement to be controlled by the external magnetic field. When the field is removed, superparamagnetic particles are no longer magnetized and have no magnetic memory^11^. However, Fe_3_O_4_ is readily oxidized to hematite (Fe_2_O_3_), changing its magnetism from superparamagnetic to canted antiferromagnet, and reducing its saturation magnetization. In any case, it would reduce the magnetic properties performance. To avoid oxidation and to protect the metal core, natural and synthetic polymers and silica have been employed to coat the magnetic particles^13^. Moreover, silica is widely used for coating magnetic nanoparticles functioning as protection against potential toxicity and aggregation. The coating not only improves biocompatibility and physicochemical stability but also enables the synthesis of multifunctional particles^14,15^. A wide range of chemical functional groups can be introduced on the coating surface to increase stability, wetting properties and binding flexibility for various applications.

Current reports indicate potential of magnetic silica nanoparticles in diagnostic application by binding, extraction and purification of biomolecules including RNA, DNA, protein, enzymes and organic small molecules^16–18^. Magnetic nanoparticles can easily separate viral RNA and DNA from complex clinical samples without need of centrifugation steps and laborious traditional organic extraction or column separation techniques^19^. Using external magnetic field nucleic acids are easily separated and recovered after binding to magnetic nanoparticles^20^. Silica-coated MNPs are broadly used to extract biological molecules, including nucleic acids. To achieve better yield of the nucleic acid separation, MNPs are functionalized by covalent binding of different ligands and polymers on their surface as for example amine, aldehydes, poly acrylic acid^21^ or APTES (3-aminopropyl triethoxysilane)^22^. MNPs with functionalized surface were used for isolation of virus nucleic acid and subsequent detection of virus by PCR for example for Zika virus^12^, Epstein-Barr virus and hepatitis virus type B^21^, SARS-CoV-2^22–24^, canine parvovirus^25^ and others^26^.

**Figure 1:**
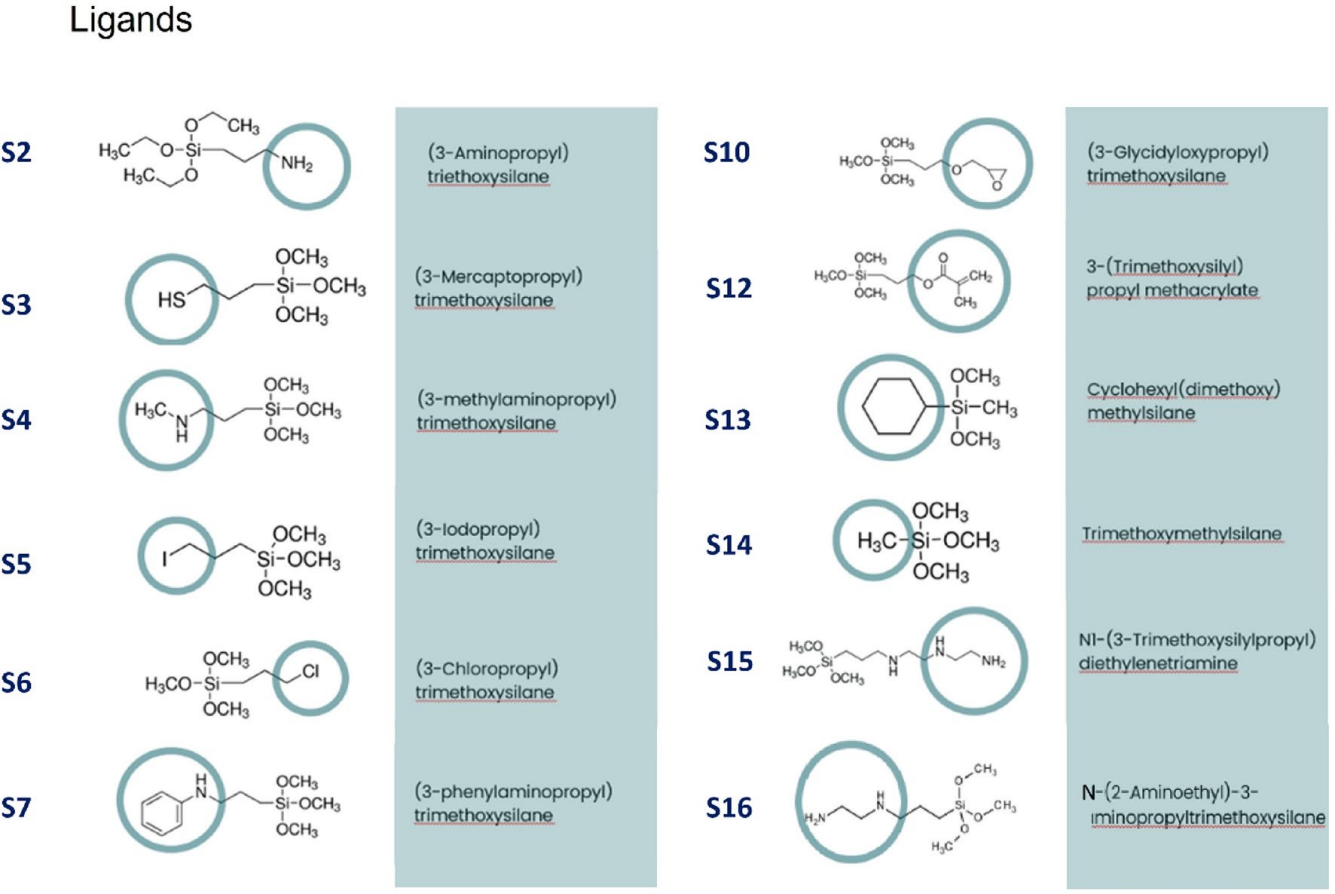
The schematic representation of **strategy A**. Modified silica-coated magnetite nanoparticles and the corresponding ligands used for the modification.

Taking advantage of our extensive experience^27–32^ in the study of core–shell magnetic nanoparticle beads, we have prepared 13 different magnetic nanoparticles systems (MNPs), the surface of which was functionalized with silane derivatives, by proposing 3 different strategies (A, B, C) for the preparation of nanoparticles. We hypothesized that the presence of specific ligands on the surface of the examined MNPs will improve yields for RNA isolation from clinical samples of infected humans or animals compared to a commercial kit. The study presents the properties of a series of MNPs with the same magnetic beads in the MNP core that have been functionalized with various silane derivatives on the outer shell. The aim of this work was the preparation and detailed characterization of different types of MNPs, ii/ screening of MNPs for the isolation of viral RNA from biological samples, namely a/ hepatitis E virus RNA from pig tissue, b/ testing of MNPs for the isolation of SARS-CoV-2 RNA from COVID-19 patient samples. In this study, we focused on the surface functionalization of magnetic silica nanoparticles and investigated their efficiency for the isolation of viral RNA in diagnostic RT-qPCR. Moreover, magnetic silica nanoparticles with porous silica layer were synthesized in order to determine the effect of porosity on viral RNA isolation. We prepared a wide range of magnetic nanoparticles, the synthesis of which consisted of three synthetic parts: 1st: synthesis of the magnetic core, 2nd: coating the magnetic core with a non-porous layer of SiO2 or oleic acid and 3th: functionalization of the surface with organic ligands (Fig. 1) or a porous layer of SiO_2_. The isolation of viral RNA was tested on the tissue from liver sample of pig infected with hepatitis E virus—HEV and on biofluid samples from patients infected by SARS-CoV-2.

## Experimental materials and methods

### Synthesis of three-layered functionalized silica-coated magnetite nanoparticles

#### First layer: magnetite Fe_3_O_4_ nanoparticles

Magnetite nanoparticles were synthesized following Bio-On-Magnetic-Beads (BOMB) procedure^33^ starting with 0.333 M FeCl_3_ (3.24 g), 0.167 M FeCl_2_ (2 g), 60 mL of 0.1 M HCl solution.

#### Second layer: non-porous SiO_2_ layer on the surface of magnetite nanoparticles

Synthesis followed BOMB procedure. The magnetite nanoparticles prepared in the previous step were prewashed in ethanol. After that 2 L of 99% ethanol, 50 mL of 25% ammonia solution and prepared iron oxide magnetic nanoparticles (~ 1.2 g) were mixed in a 2.5 L boiling flask equipped with the condenser and stirred using magnetic stirrer (400 rpm). The solution was heated up to 80 °C. Then 45 mL of TEOS was added to the mixture under constant stirring and incubated for another 30 min. Subsequently, 400 mL of ddH_2_O was added to the solution. The reaction was left to proceed overnight. Then the reaction mixture was cooled down to room temperature (RT). The silica coated magnetic nanoparticles were separated using strong neodymium magnet. Nanoparticles were washed twice with pure water, twice with pure ethanol and again with pure water until the pH of the solution became neutral. The prepared nanoparticles were denoted as sample **S1**.

#### Third layer: functionalized silica coated magnetite nanoparticles

##### Strategy A: functionalization by organic ligands

Magnetite nanoparticles coated with silica (Fe_3_O_4_@SiO_2_, sample **S1**) were modified by several organic ligands presented in Fig. 1. Typically, 1 g of sample **S1** was dispersed in 70 mL of anhydrous toluene in boiling flask and mixed with 1.5 mL of the respective organic ligand, shown in Fig. 1 and refluxed under N_2_ overnight. Then the reaction mixture was cooled down to RT and the obtained product was separated using strong neodymium magnet. Nanoparticles were washed three times by toluene, three times by ethanol and three times by water. The product was dried in an oven at 45 °C for 2 h and then the product was air dried at RT. The procedure was identical for all ligands used. The prepared samples with organic ligands were denoted as samples **S2–S16**. For the final comparison in the study, only the samples **S2**, **S3**, **S4** and **S6** were selected since they exhibited the highest efficiency with respect to the application potential.

##### Strategy B: functionalization by porous silica

**Figure 2:**
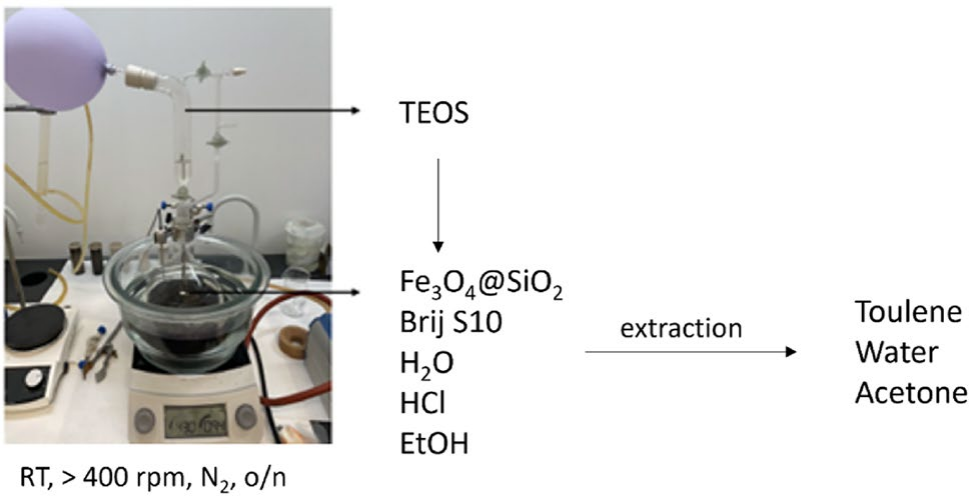
The schematic representation of functionalization B—porous silica. A picture of the experimental apparatus used in the synthesis of eMNPs.

For the synthesis of porous silica coated magnetite nanoparticles the silica coated magnetite nanoparticles prepared by the procedure described above (sample **S1**) were used. In a typical procedure 2.5 g of sample **S1** was dispersed in a solution of 8 g Brij S10 dissolved in 2 L of deionized water and 160 g of HCl (2 M). The solution was homogenized for 30 min to form a uniform dispersion. Then, 10 g of TEOS was added dropwise to the dispersion with continuous stirring and the reaction proceeded overnight. The product was collected with a strong neodymium magnet and washed repeatedly with ethanol and water to remove nonmagnetic by-products. Finally, the pore-generating agent (Brij S10) was removed by extraction or calcination. Half of the synthesis yield was removed by extraction in toluene under reflux at 110 °C for 2 h. The second half of the product was calcined in the oven in airflow at 550 °C. The sample was heated to 550 °C with a heating rate 3 °C/min and the sample was held at this temperature for 16 h. Subsequently, the sample was cooled down to room temperature. The calcined sample was washed with ethanol, magnetically separated and the product was dried in an oven at 110 °C for 24 h. Samples were denoted: **S21** (extraction) and **S22** (calcination). The experimental setup used in the synthesis is shown in Fig. 2.

#### Synthesis of hydrophobic porous oleic acid coated magnetite nanoparticles

##### First and second layer: oleic acid coated magnetite nanoparticles

Hydrophobic magnetite nanoparticles were synthesized by one-pot chemical co-precipitation method reported previously^34^. Deionized water was purged with nitrogen gas for 10 min. Then, 4.8 g of FeCl_3_·6 H_2_O, 2.0 g of FeCl_2_·4 H_2_O, and 0.85 mL of oleic acid (OA) were added to 30 mL of deionized water under nitrogen atmosphere with vigorous stirring. The mixture solution was heated to 90 °C. Then, 20 mL of ammonium hydroxide (14 wt%, 11.2 mL NH_4_OH + 8.8 mL H_2_O) was added rapidly to the solution, and it immediately turned black. The reaction was kept at 90 °C for 2.5 h and then allowed to cool to room temperature. The black precipitate was collected by magnetic decantation and several times washed with methanol (yield: ~ 2.25 g).

##### Third layer: porous oleic acid coated magnetite nanoparticles

***Strategy C****: functionalization by porous silica* Typically, 2.25 g Fe_3_O_4__OA nanoparticles were dispersed in 2 L deionized water under bath ultrasonication for 30 min. Subsequently, 4 g CTAB was added to the dispersion with vigorous stirring for 1 h. Then, 12 mL TEOS was dissolved in 600 mL of EtOH and dropwise added to the reaction medium during 5 h. After adding of all TEOS, the reaction proceeded overnight. Then, the resulting suspension was magnetically separated and washed with 20% ethanol three times. Finally, the pore generating agent CTAB was removed by an ethanolic solution of ammonium nitrate (NH_4_NO_3_, 6 g/L, 60 mL) under reflux at 60 °C for 2 h. Sample was magnetically separated and washed with water. The product was dried in an oven at 110 °C for 24 h. Sample was denoted as sample **S23**.

### TEM, SEM, XPS and XRD measurements

The basic evaluation of the morphology and size of particles was carried out by a transmission electron microscope FEI Tecnai G^2^ 20 equipped with a thermionic cathode (LaB_6_) and an acceleration voltage of 200 kV was applied. Chemical mapping, SAED analysis and TEM imaging in high resolution (HRTEM) were performed on an FEI Tecnai TF20 X-twin microscope equipped with a field emission gun and an EDAX detector and operated at 200 kV. The EDX maps were recorded in scanning TEM (STEM) mode by using the TIA software. The morphologies and structures of prepared samples were characterized also by field emission scanning electron microscopy (JEOL JSM-IT700HR) with an accelerating voltage of 15 kV). Samples for FE-SEM observations were prepared on carbon-coated copper grids. EDX (JEOL EX-74212U4L2Q) analysis were used to confirm the presence of elemental Fe, O and Si atoms in prepared samples.

For our high-resolution X-ray photoelectron spectroscopy (XPS) we used an Al anode operating at 200 W and the SPECS PHOIBOS 100 analyzer, with a base pressure of 10^–8^ mbar. The samples were attached to a molybdenum sample holder using conductive carbon tape.

X-ray diffraction (XRD) experiments were measured in reflection mode with Bragg–Brentano parafocusing geometry using a Rigaku Ultima IV multipurpose diffractometer. X-ray lamp with Cu-Kα_1,2_ radiation (λ = 0.15406 nm) was used. Powder samples were put on a glass sample holder. Diffracted photons were collected using a D/teX Ultra high-speed, position-sensitive detector system by scanning 2θ range from 10° up to 90° with the step size of 0.02°. To ensure reliable diffracted intensities each sample was spinning at 30 rpm during collecting XRD data.

### Infrared spectroscopy

The infrared spectra of the studied experimental samples were measured using Nicolet 6700 FT-IR spectrometer at ambient temperature and using the KBr technique in the wavenumber range of 4000–400 cm^−1^. Samples were mixed with dry KBr (preheated at 600 °C) in a mass ratio 100:1. The measuring settings were 64 scans for a single spectrum with 4 cm^−1^ resolution^28^.

### N_2_ sorption/desorption measurements

The porosity of prepared porous samples **S21**, **S22** and **S23** was estimated using Quantachrome NOVA 1200e adsorption analyzer at − 197 °C by nitrogen adsorption/desorption measurements^27^. Each sample was first outgassed at 110 °C for 12 h under vacuum. A standard Brunauer–Emmett–Teller (BET) theory was used for calculations of the specific surface area (*S*_*BET*_) for samples **S21**, **S22** and** S23**.

### Magnetic measurements

SQUID based magnetometer MPMS 5XL (Quantum Design) was used for performing of magnetic measurements. Powder sample was encapsulated in a gelatine capsule and fixed in the plastic straw^44,45^. Isothermal magnetization data were obtained as follows. The sample was cooled in the absence of the applied magnetic field down to targeted temperature. Then the magnetization vs applied field *M(H)* loop was recorded up to ± 7 T. Further, the temperature was elevated, fixed to a higher value (up to 300 K), and another *M(H)* loop was taken. ZFC magnetization vs temperature (1.8–330 K) data were obtained after cooling the sample from 330 K in zero applied magnetic field down to 2 K. Subsequently, static magnetic field was applied and magnetization has been recorded while heating the sample to 330 K. The FC magnetization data were collected during cooling the sample in the same applied field back to 2 K^44,45^.

### Application of magnetic nanoparticles for isolation and detection of HEV in pig liver tissue

#### Isolation of viral RNA from pig liver sample

To verify the quality of experimentally prepared magnetic nanoparticles (eMNPs), the screening on a clinical sample obtained from the liver tissue of naturally HEV infected pig was used. Total RNA was obtained from liver sample using commercially available MagMAX™—96 Total RNA Isolation Kit (Applied Biosystems, Vilnius, Lithuania) which was designated for the isolation of total RNA from animal tissue. The magnetic beads in commercial kit (comMNPs) were replaced by our eMNPs and all other steps were identical with those described for a kit. In principle, the procedure started with homogenization of approximately 5 mg of frozen liver sample (as recommended in kit instruction). The sample was disrupted in quanidin thiocyanate-based solution to solubilize cellular membranes and inactivate nucleases^35^. After homogenization, the samples were mixed with eMNPs to enable RNA to bind to the surface of magnetic beads (4 min/RT). The eMNPs with bound RNA was captured by a magnet located in magnetic stand (3 min/RT) and then washed with two wash solutions from the kit to remove all cell components, including proteins. To destroy DNA in the solution of isolated RNA, the treatment with DNase was used in the final RNA clean-up step. Finally, RNA from eMNPs was eluted into 50 µl elution buffer with low ionic strength and stored at – 80 °C before next experiments. All details of isolation procedure are described in manufacturer instructions for the kit. The concentration and purity of isolated RNA was measured by absorbance A260/280. In all RNA isolation experiments, the concentration of replaced magnetic beads was adjusted to 130 mg/mL for each sample of eMNPs. Optimal concentration of eMNPs, comparable to the commercial kit was determined by measuring of the magnitude of the magnetic moment and the saturation magnetization of the magnetic moment using a SQUID device. The higher mass of the used eMNPs is due to the additional layer in the eMNPs in comparison to the comMNPs. The liver sample from naturally infected pig has been taken in slaughter-house according to Council Regulation (EC) No 1099/2009 of 24 September 2009 on the protection of animals at the time of killing as well as Commission Implementing Regulation (EU) 2019/627 of 15 March 2019 laying down uniform practical arrangements for the performance of official controls on products of animal origin intended for human consumption in accordance with Regulation (EU) 2017/625 of the European Parlament and of the Council and amending Commission Regulation (EC) No 2074/2005 as regards official controls.

#### Detection of HEV RNA by one-step RT-qPCR

All liver samples isolated with eMNPs as well as with comMNPs were screened for HEV by one-step real-time RT-qPCR assay using the iTaq Universal Probes One-Step Kit (Bio-Rad Laboratories, Inc., USA). The one-step RT-qPCR reaction mixture was composed of 2 µL of isolated RNA, 10 µL of iTaq Universal Probes Reaction Mix, 0.5 µL of iScript Reverse Transcriptase, primers (JVHEVF, JVHEVR) at concentration of 250 nM, probe (JVHEV-P) at concentration of 100 nM^36^, and filled with molecular biology grade water (Merck, GmbH, Germany) to the final 20 μLvolume. The one-step RT-qPCR reaction was carried out at 50 °C for 10 min, 95 °C for 3 min, and 45 cycles at 95 °C for 15 s and 55 °C for 30 s. The thermal profile and amplification of DNA was performed using CFX Opus Real-Time PCR Systems (Bio-Rad Laboratories, Inc., USA). The efficiency of RNA isolation by eMNPs and the detection of HEV by one-step RT-qPCR were compared to values obtained by commercial kit. To obtain more objective results of the concertation of RNA and C_t_ value of RT-qPCR, the values are presented as the average of 5 experiments with the same sample. The standard deviation was calculated automatically by MS Excel.

### Application of magnetic nanoparticles for isolation and detection of SARS-CoV-2 in biofluid/swab samples

#### Isolation of viral RNA from nasopharyngeal swabs

Selected eMNPs, namely **S1**, **S3**, **S6**, **S21**, **S22** and **S23** were tested for isolation SARS-CoV-2 RNA from nasopharyngeal swabs of COVID-19 affected patients. The isolation of the viral RNA was accomplished by commercially available MagMax™ Viral RNA isolation Kit (Applied Biosystems, Vilnius, Lithuania), which is designated for the isolation of RNA and DNA from biofluid samples. The kit uses the same principle for RNA isolation as used for the isolation of HEV from tissue; i.e. the lysis of cell material and binding of RNA to magnetic beads in guanidin thiocyanate-based buffer. Isolation of RNA was carried out according to the manufacturer’s instructions; the replacement of comMNPs by our eMNPs was identical as described for the isolation of viral RNA from the liver tissue. Clinical samples for RNA isolations consisted of anonymized swab samples from the nasopharynx and throat of patients infected by SARS-CoV-2. Each clinical sample was simultaneously analyzed also by using the comMNPs.

#### Detection of SARS-CoV-2 RNA by one-step RT-qPCR

SARS-CoV-2 RNA was detected using a commercial IVD-certified COVID-19 Real Time Multiplex RT-PCR Kit (Labsystems Diagnostics Qy, Vantaa, Finland), which was widely used for identification of COVID-19 patients in Slovakia. This one-step multiplex kit is used for the amplification and detection of three SARS-CoV-2 genes (*ORF1ab*, *E*, *N*) together with one human gene as an internal control (IC). Amplification of individual genes was monitored in four fluorescent channels (gene—dye/channel): E gene—FAM, ORF1ab gene—HEX, N gene—TEXAS RED, IC—CY5. The composition of the reaction mixture and the amplification profile was carried out according to the manufacturer's instructions, in the thermocycler CFX96 Touch Real Time PCR Detection System (Bio-Rad, Hercules, USA). The course of amplification was monitored and evaluated with the Bio-Rad CFX Maestro 1.1 software. RNA samples isolated from the same clinical material using different eMNPs were always analyzed simultaneously and evaluated at the same setting of threshold for the individual fluorescence channels.

### Ethics declarations

We declare that all experimental protocols were approved by a named institutional and/or licensing committee.

Animal approval is granted by Council Regulation (EC) No 1099/2009 of 24 September 2009, Commission Implementing Regulation (EU) 2019/627 of 15 March 2019, Regulation (EU) 2017/625 of the European Parlament and of the Council and amending Commission Regulation (EC) No 2074/200, State Veterinary and Food Administration of the Slovak Republic and by Ethics Commission of the University of Veterinary Medicine and Pharmacy in Kosice, Slovakia.

For human, Certificate of Accreditation (No. M-001) for analyses of SARS-CoV-2, Ethics Commission of the synlab slovakia s.r.o Limbova 5, 831 01, Bratislava, Slovakia.

We declare that all methods were carried out in accordance with relevant guidelines and regulations.

For animals, the protocol for collection of clinical samples followed the guidelines stated in the Guide for the Care and Use of Animals (protocol number 3323/16–221/3) which was approved by the State Veterinary and Food Administration of the Slovak Republic and by Ethics Commission of the University of Veterinary Medicine and Pharmacy in Kosice, Slovakia.

For human, all experimental protocols were approved by Ethics Committee, synlab slovakia s.r.o. The use of the experimental protocol is in line with the Code of conduct. The samples for SARS-CoV-2 analyses were de-identified according to laboratory rules.

We declare that the informed consent for the experimental SARS-CoV-2 analysis of the sample was obtained from all subjects and/or their legal guardian(s).

We declare that the study is reported in accordance with ARRIVE guidelines.

## Results

### Structure and morphology

**Figure 3:**
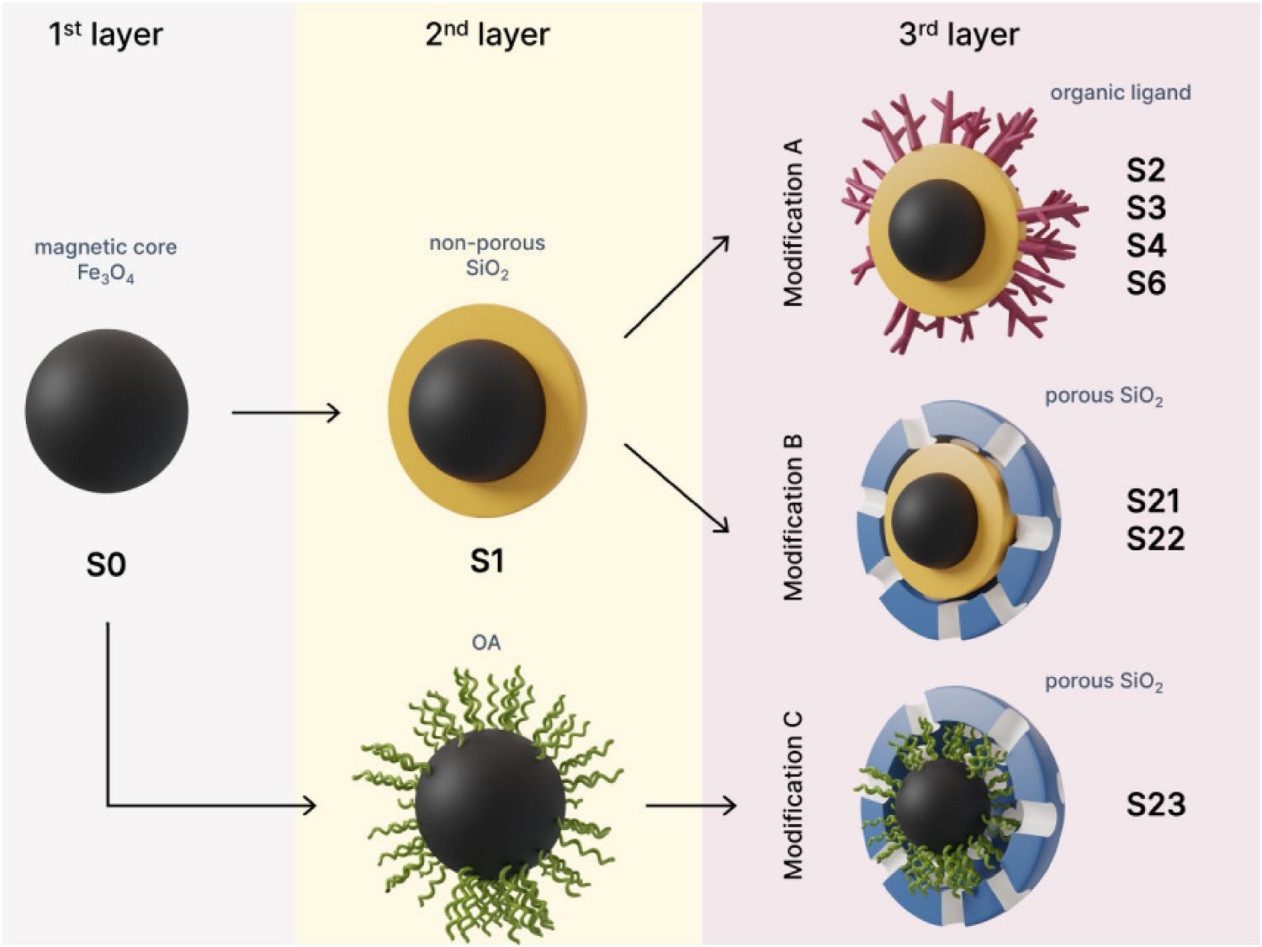
Schematic representation of three different **strategies A**,** B**, and **C** for preparation of the experimental samples of magnetic nanoparticles (eMNPs).

Three different strategies were used for preparation of structurally and functionally modified magnetic nanoparticles. The aim was to assess the effect of functional group modification and surface modification on the effectiveness of RNA separation; see schematic representation on Fig. 3.

**Figure 4:**
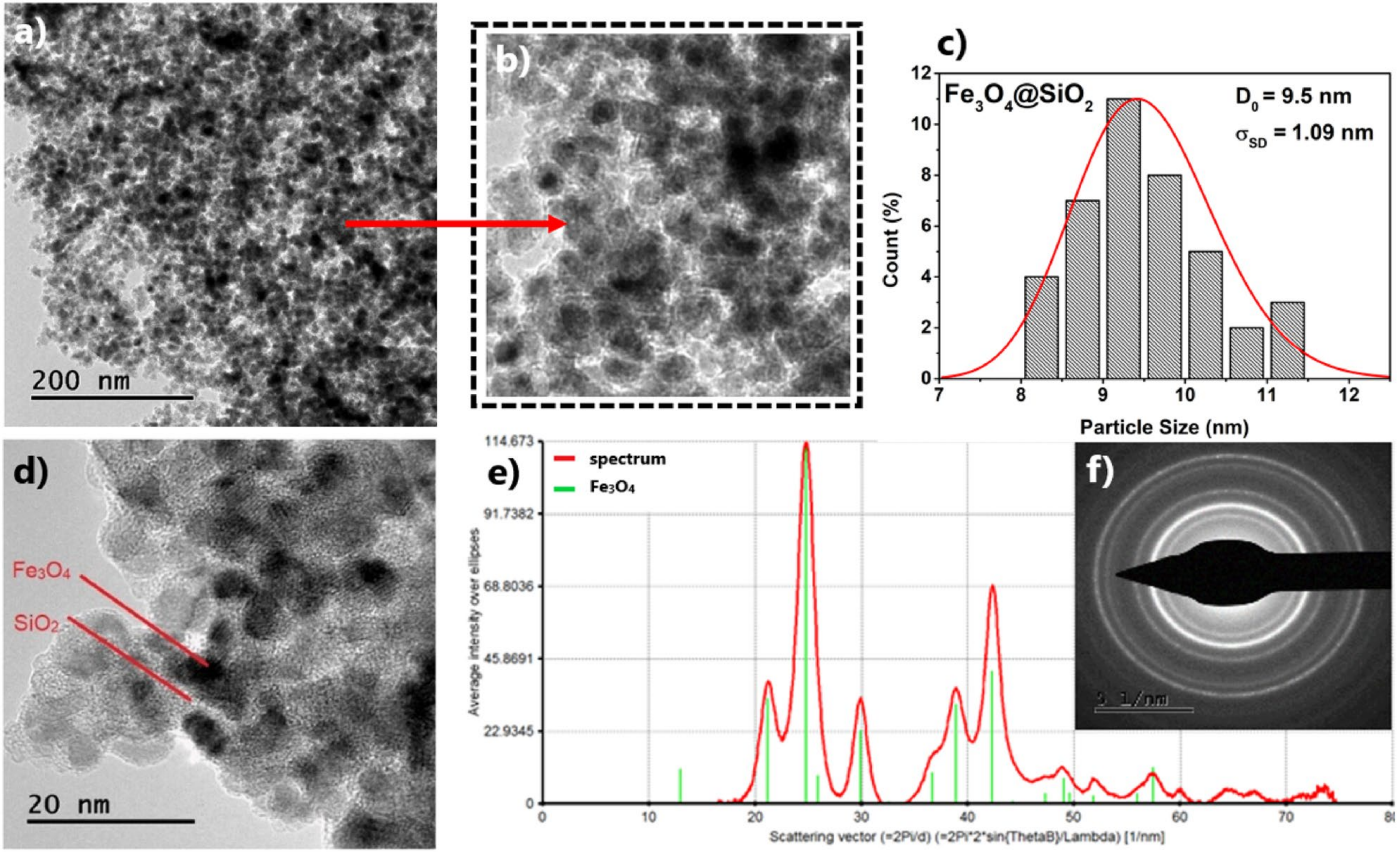
Advanced characterization of sample **S1**. (**a**, **b**, **d**) Representative TEM micrographs, (**c**) size distribution of silica-coated nanoparticles, and (**e**, **f**) electron diffraction pattern and analyzed phase composition.

**Figure 5:**
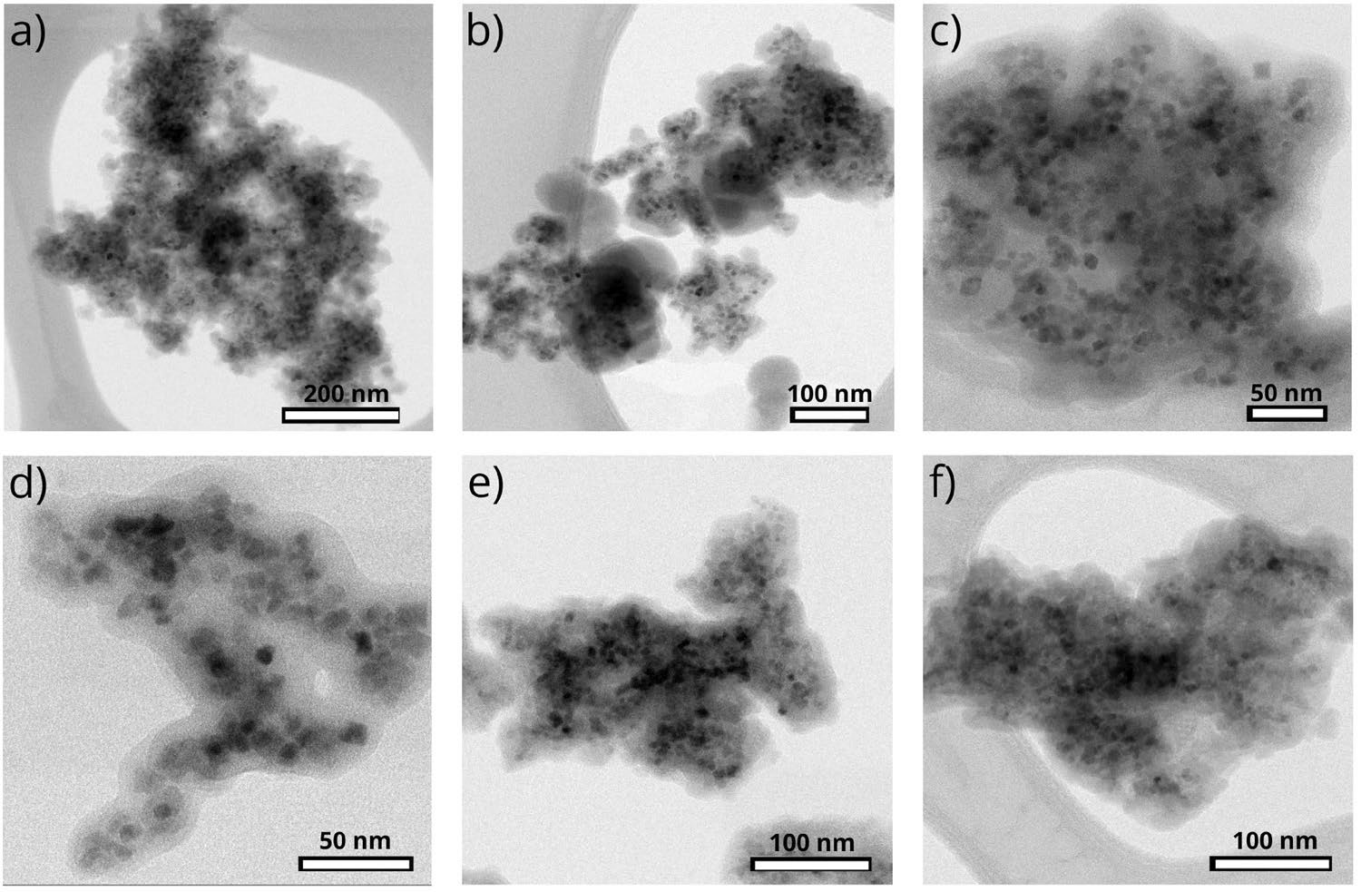
Representative TEM micrographs of samples prepared by strategies A, B, and C where the same magnetic core **S1** is present. (**a**) Sample S2, (**b**) sample S3, (**c**) sample S4, (**d**) sample S6, (**e**) sample S21, (**f**) sample S23.

The morphology of the silica-coated magnetic nanoparticles that were used as base for next surface and functional modification (sample **S1**), is shown in TEM images (Fig. 4a,b). Nanoparticles are composed of magnetic core with the diameter around 10 nm and the surrounding layer of SiO_2_ with a thickness of about 5 nm (Fig. 4c,d). Electron diffraction pattern (Fig. 4f) and its analysis (Fig. 4e) confirm the presence of pure cubic iron-oxide phase corresponding to Fe_3_O_4_. The TEM micrographs of the surface modified samples are provided in Fig. 5. The average TEM size of an iron-oxide core corresponding to the sample **S2** (the representative of the organic ligand modified series **S2–S16**) and **S21** (the representative of porous silica modification series **S21**, **S22**) have been determined to *D*_*TEM*_ ~ 8 nm, (Figs. C1 and C2 in Supplementary Information). The value (although slightly lower) is in accordance with the untreated beads **S1**.

Electron diffraction by TEM microscopy was used as evidence for the presence of a pure cubic Fe_3_O_4_ phase in all **S2–S23** samples studied. Despite the fact that the same Fe_3_O_4_ magnetic core (from the same synthesis) was always used in the synthesis of the coated particles in **strategies A**, **B** and **C**, we wanted to investigate whether partial oxidation of the magnetite core occurs during the surface modification process. Our results (see Fig. C2 Supplementary Material) confirm that in all surface modified samples with both an organic shell (**strategy A**, samples **S2–S16**) and an inorganic shell (**strategies B** and **C**, samples **S21–S23**), both the phase composition and the size of the magnetic core are the same in all systems.

**Figure 6:**
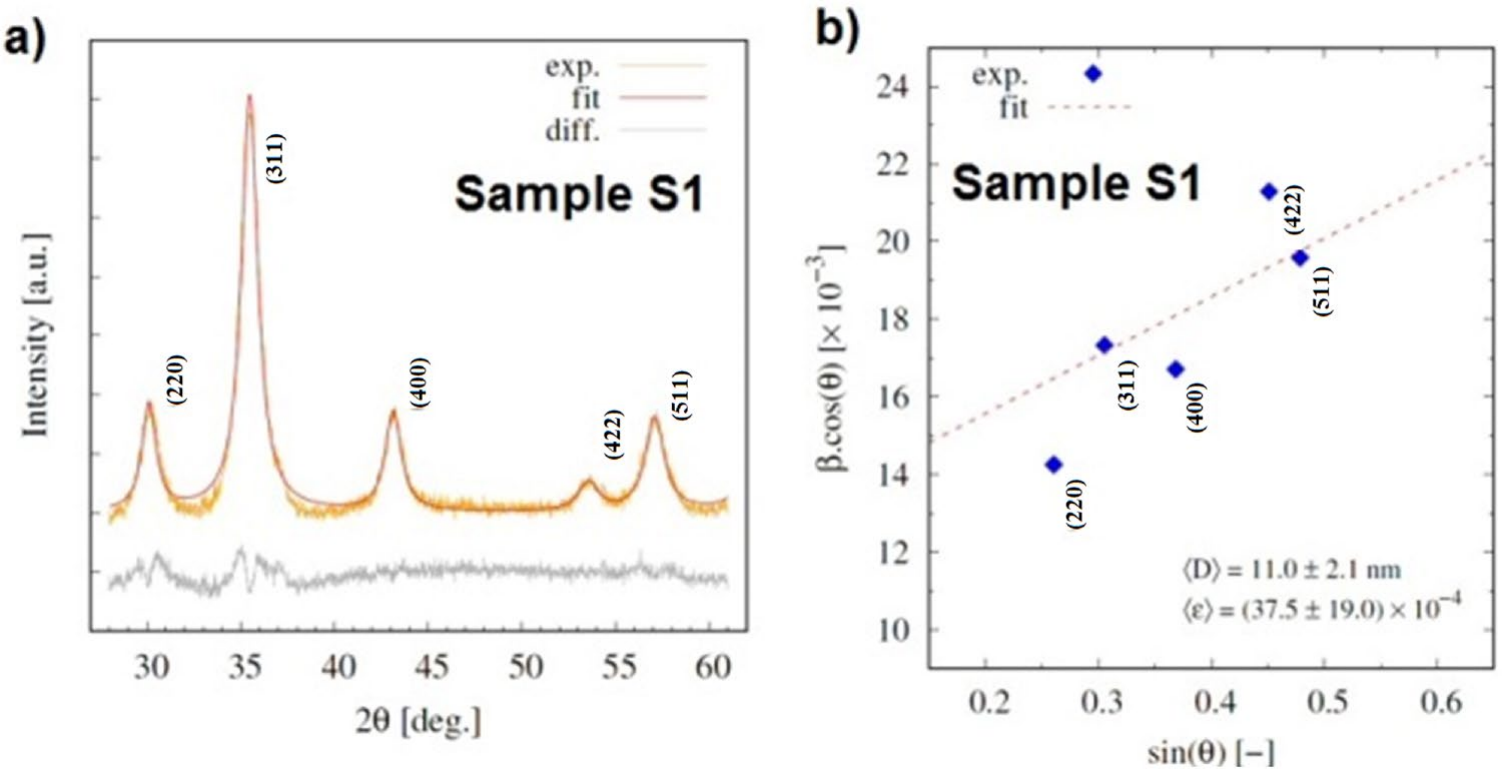
XRD data of sample **S1**. (**a**) Experimentally determined XRD pattern and calculated model (red line) based on the structural model of cubic phase Fe_3_O_4_. Vertical tics mark positions of Bragg peaks corresponding to cubic Fe_3_O_4_ phase. Background contribution was subtracted from the XRD profile, (**b**) analysis of the diffraction pattern by the Williamson-Hall method.

XRD data revealed the presence of the cubic iron-oxide phase (Fig. 6). In order to confirm the presence of magnetite (Fe_3_O_4_) instead of possible maghemite (γ-Fe_2_O_3_) phase, X-ray photoelectron spectroscopy (XPS) measurements were performed and discussed later. The Williamson-Hall analysis applied to the sample consisting of magnetic beads (**S1**) before its surface modifications is shown in Fig. 6b. From the linear regression fit, the average crystallite size *D*_*XRD*_ = 11.0 ± 2.1 nm has been determined. When compared to TEM size, a slightly larger grain size (although still within the experimental error) can be attributed to the presence of a small fraction of larger crystallites in the sample observed by TEM. The high value of microstrain ε = 3.75 ± 1.9 × 10^–3^ established with the aid of the Williamson–Hall analysis is characteristic of applied synthesis method for the iron-oxide cores’ fabrication. As it will be discussed later, microstrain has crucial impact on the magnetic properties of the iron-oxide NPs. The rest of the examined sample series have been analyzed by employing the Scherrer equation (Supplementary Information) and the obtained average crystallite sizes are listed in the Table 1. From the mutual comparison of XRD patterns of the whole sample series (Supplementary Information, Figs. C3, C4), one can draw several conclusions. The surface modification of the magnetic beads (**S1**) by organic ligands (**S2**–**S16**) has not affected the diffraction pattern. On the other hand, the presence of significant fraction of amorphous silica in the samples **S21**, **S22**, **S23** is clearly evident from the maximum at 2θ ~ 22°.

**Table 1:** The comparison of structural and magnetic characteristics of the studied samples.

Sample	D_XRD_ (nm)	D_SEM_ (nm)	Zetapotential (mV) at pH 7	D_mag_ (nm)	Ms (5 T, 300 K) (emu/g)	S_BET_ (m^2^/g)
S1	11.0	19.8	− 29.3	13	33.5	85,7
S2/APTES	10.5	59.6	+ 27.9	14	10.5	37,8
S3/MPTMS	13.5	66.5	− 21.6	14	9.0	22,4
S4/MAPTMS	10.5	78.5	+ 8.2	13	–	15,8
S6/CPTMS	11.3	98.7	− 25.8	13	8.9	23,7
S10/GOTMS	13.0	77.3	− 22.4	13	–	21,2
S12/MMSP	13.1	77.4	− 27.1	15	18.5	29,9
S13/CHMMS	13.4	86.5	− 19.2	13	–	23,8
S15/MPDETA	12.6	89.3	+ 18.8	12	–	26,1
S16/AEAPTMS	13.7	89.5	+ 10.7	14	–	18,7
S21	9.3	93.9	− 28.7	12	2.4	553
S22	8.4	92.9	− 25.4	13	2.2	727
S23	8.5	101.3	− 2.8	12	–	170

**Figure 7:**
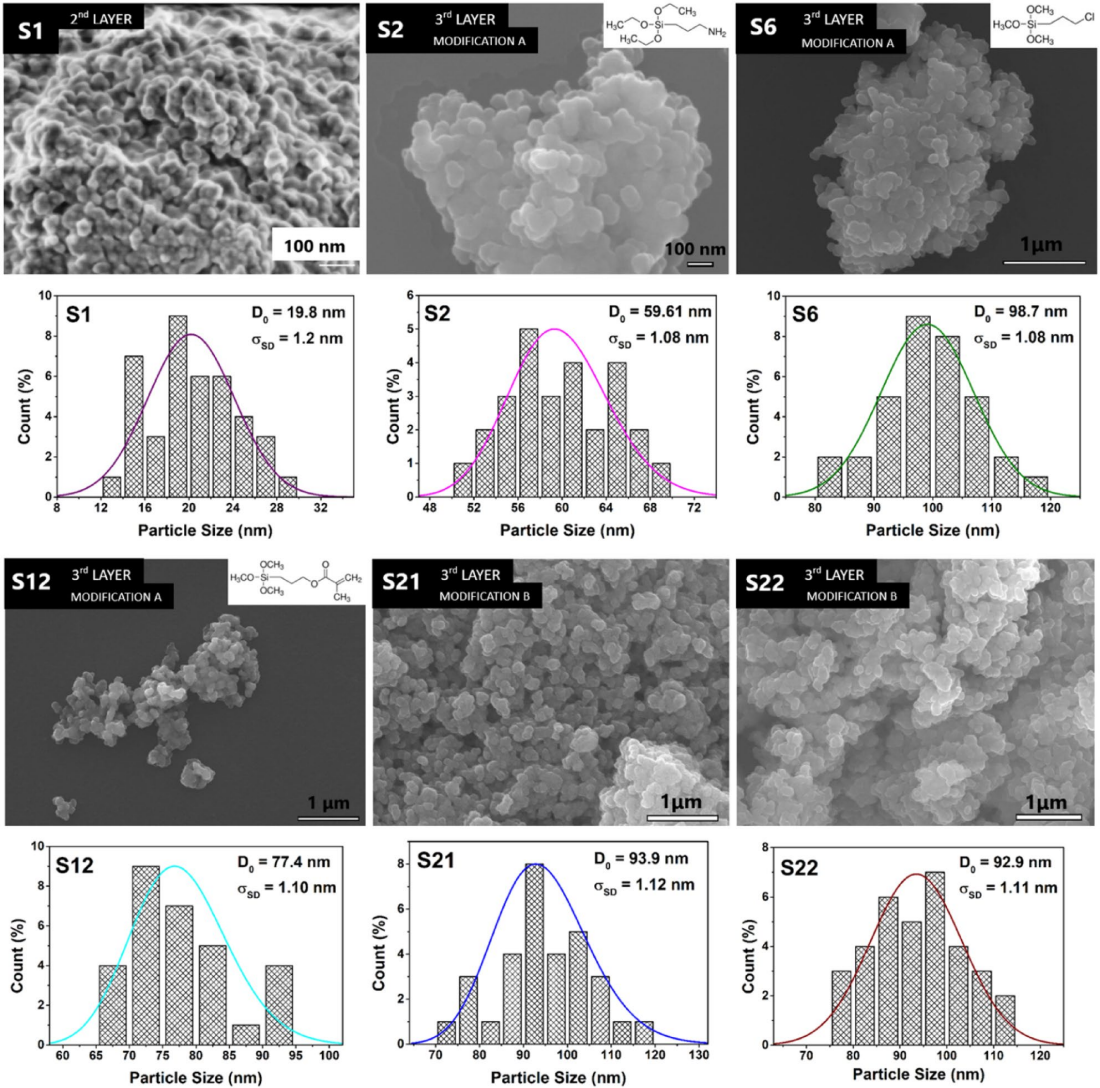
SEM micrograph of selected studied samples **S1**, **S2**, **S6**, **S12**, **S21**, **S22** and the size histograms derived from SEM micrographs.

The morphology of studied samples was analyzed also by the SEM. Micrographs of selected samples obtained from SEM presented on Fig. 7 showed the morphology and size of nanoparticles that are spherical shaped and apparently aggregated into larger clusters. For this reason, the average SEM sizes that are presented in the Table 1 and the size distributions sown in the Fig. 7 correspond to the aggregates rather than individual nanoparticles. The sample **S1** represented basic nanoparticle coated only with SiO_2_ shell, while the samples **S2** to **S16** consisted of Fe_3_O_4_/SiO_2_ (sample **S1**) core–shell nanoparticles coated with various ligand. Different ligands contributed to the different physicochemical properties and final overall sizes of modified nanoparticles. All iron oxide cores were prepared according to the same synthesis protocol which determined their size, and were coated with silica shell, and subsequently coated with ligands. Nanoparticles without ligand layer (sample **S1**) exhibit total average size *D*_*SEM*_ ~ 20 nm. In the rest of the samples (**S2–S16),** the size of eMNPs differed depending on the ligand. Since the ligand groups differed in their chemical composition, molecular weight and functional groups thickness of the third shell varies accordingly. For example, CPTMS ligand coated nanoparticles (sample **S6**) formed a thicker ligand layer, of about 40 nm and in contrary, third shell of the sample coated with APTES ligand (sample **S2**) was ~ 20 nm thick. The particles with the largest size have the third layer formed by an inorganic capping of porous SiO_2_ (samples **S21** and **S22**), as can be seen from the comparison in the Fig. 7 and in the Table 1.

**Figure 8:**
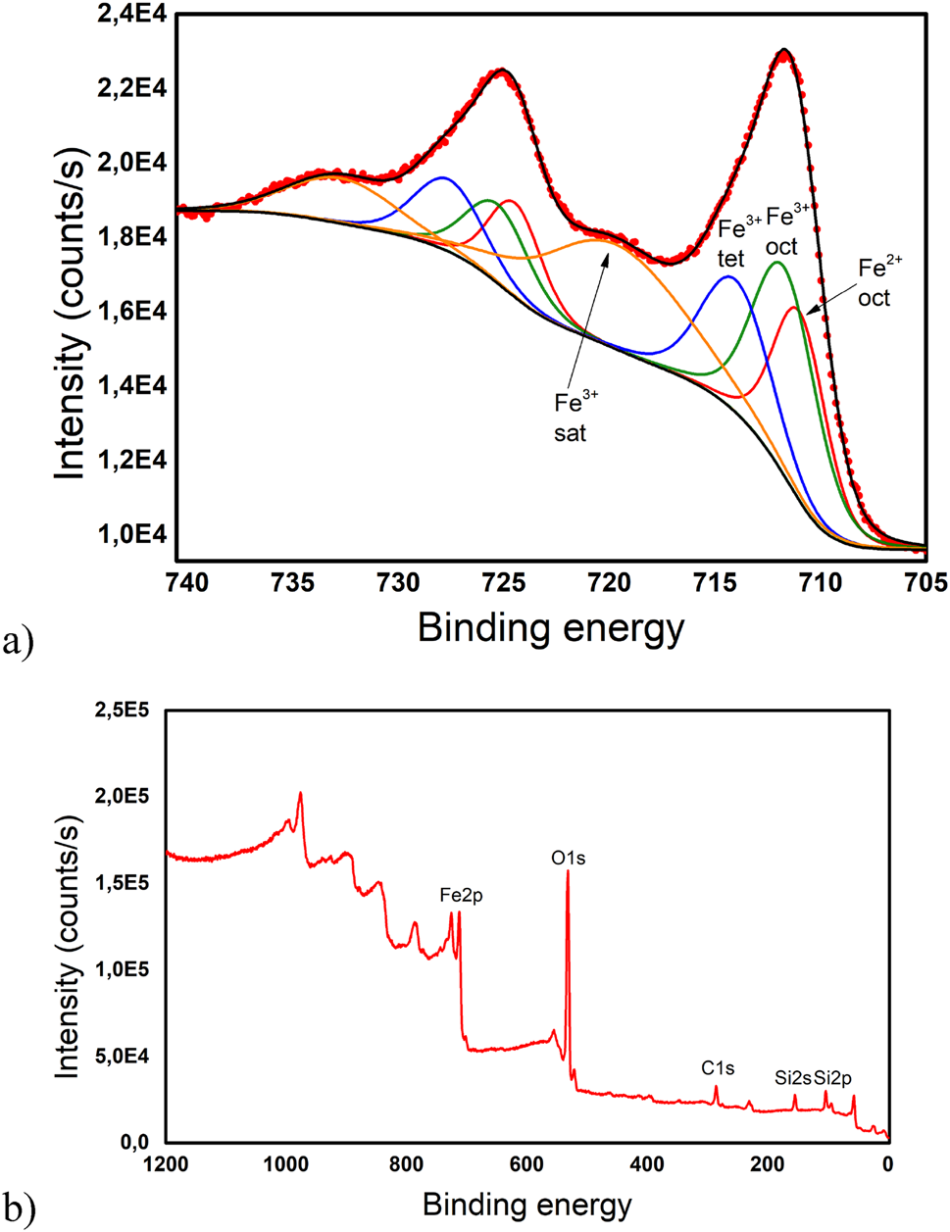
(**a**) The high resolution Fe 2*p* XPS spectrum of Fe_3_O_4_@SiO_2_, (**b**) The XPS survey of Fe_3_O_4_@SiO_2_.

In the Fe_3_O_4_ structure, the iron atoms are present in two oxidation states, Fe^2+^ and Fe^3+^. Both cations occupy the octahedral sites (oct.), while Fe^3+^ cations also occupy the tetrahedral sites (tet.) in the inverse spinel structure, Fig. 8. This creates different electron environments distinguishable by X-ray photoelectron spectroscopy (XPS). The measured Fe2p spectrum is shown in Fig. 8a. We found three components that fit the main Fe 2*p* 3/2 peak (on the right), corresponding to Fe^2+^ (oct) with binding energy 710.99 eV, Fe^3+^ (oct) at 711.7 eV and Fe^3+^ (tet) at 714.02 eV. We identified the peak at 719.71 eV as the Fe^3+^ satellite peak. The same set of components fits the Fe 2p1/2 peak, with half the area of the corresponding Fe 2*p* 3/2 components. The spin–orbit splitting is 13.48 eV for Fe^2+^ (oct) and 13.6 eV for both, Fe^3+^ (oct) and Fe^3+^ (tet). The Fe^2+^/Fe^3+^ ratio 0.45 was determined from the Fe 2*p* 3/2 peak. The fitting values agree with literature^37,38^. The spectrum in Fig. 8b reveals the main peaks of the sample constituents Fe, Si, and O. The extra peaks corresponding to C and Mo emerge from the adventitious carbon and the molybdenum sample holder.

The Zeta potential (ZP) and hydrodynamic particle size were measured to determine the biocompatibility, colloidal stability, and suitability of prepared nanoparticles for introduction into living organisms. Generally speaking, nanoparticle dispersions in water showing ZP magnitude ~ 20 mV (and larger) are considered as stable^39^. The complete sample series has been examined with respect to the stability at the same conditions (in pure water at neutral pH = 7 and temperature 25 °C), while ZP of selected samples is presented in the Table 1. Apparently, the largest values of negative ZP have been determined for the samples with the silica outer layer (**S1**, **S21**, **S22**). However, nanoparticles modified by specific organic ligands have also exhibited significantly large ZP values above 20 mV in absolute magnitude (**S2**, **S3**, **S6**, **S10**, **S12**). Almost all of the samples show negative ZP at pH 7, while positive values have been determined only for the group of samples modified by the ligands containing amine groups (**S2**, **S4**, **S15**, **S16**).

### Infrared spectroscopy analysis

**Figure 9:**
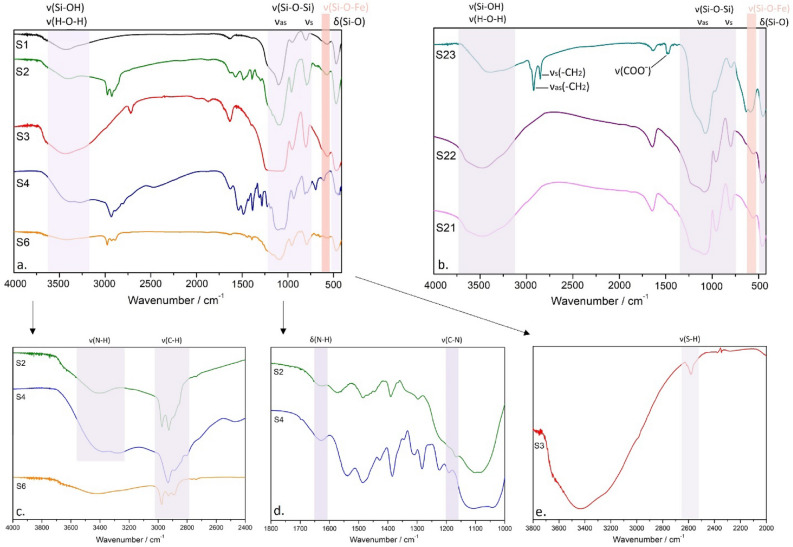
The infrared spectra of the selected studied eMNPs samples. (**a**, **b**) full spectral region, (**c**–**e**) magnified significant areas.

Figure 9 shows the FTIR spectra of selected sample **S1**, ligand modified samples **S2–S4** and **S6** and mesoporous samples **S21–S23**.

In all spectra, the absorption bands at 3650–3200 cm^−1^ correspond to Si–O–H stretching vibration. The spectra show the absorption bands near 500 cm^−1^ which are assigned to Fe–O stretching mode. The all spectra show the Si–O–Si stretching vibration at 1080–1070 cm^−1^. The existence of SiO_2_ layers in the all spectra can be seen by the Si–O–Si stretching vibration at 1080–1070 cm^−1^ as well as the Fe–O–Si stretching vibration at 1250–1050 cm^−1^. The data strongly suggest that the Fe_3_O_4_ nanoparticles were successfully coated with SiO_2_ layers. The organic ligands modified on the silica surface in samples **S2**–**S6** were confirmed by presence of the small absorption peaks at 3000–2900 cm^−1^ corresponding to C–H vibrations.

### Nitrogen adsorption/desorption measurements

**Figure 10:**
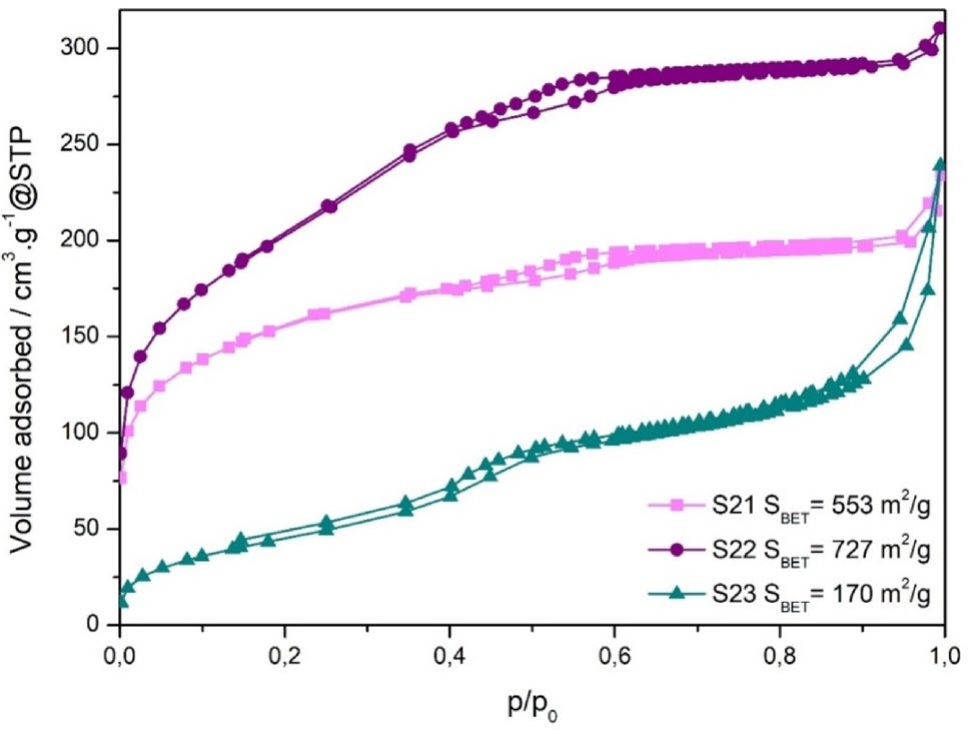
Nitrogen adsorption/desorption isotherms of the porous samples **S21**, **S22** and **S23**.

Since the samples **S1–S16** are non-porous they exhibit the negligible surface areas compared to porous systems (Table 1). The samples **S21**, **S22** and **S23** contain porous silica layer, which was confirmed by nitrogen adsorption/desorption isotherms (see Fig. 10). The uptake at low p/p_0_ is associated with the filling of micropores. A small hysteresis loop in the relative pressure range 0.2–0.6 was observed, confirming mesoporous feature of the samples. The isotherms can be classified as type IV according to the IUPAC classification^40^. However, the pores in the samples are not ordered, like e.g., in SBA-15 silica, but mesoporosity is disordered. H4 type hysteresis loop is typical for particles with internal voids of irregular shape and broad size distribution. Mesoporous character of the samples **S21**, **S22** and** S23** was also reflected by much larger surface areas in comparison with samples **S1–S16**.

### Magnetic properties measured in dc external magnetic field

**Figure 11:**
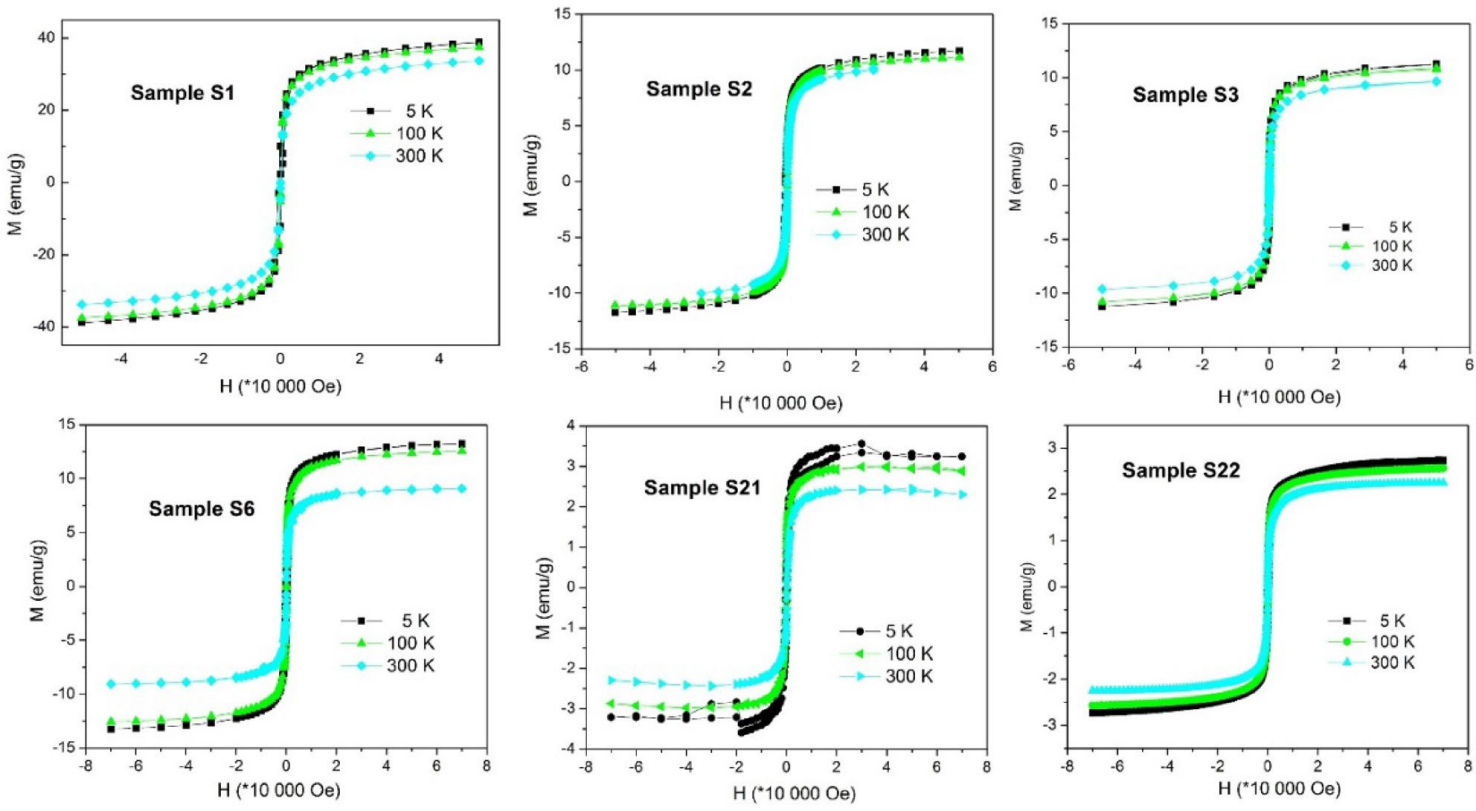
Magnetization isotherms *M(H)* measured at temperatures of 5 K, 100 K and 300 K for selected studied samples.

Magnetic isotherms of selected samples were measured at different temperatures of 5, 100 and 300 K and are shown in Fig. 11. The nanoparticles exhibited superparamagnetic behavior, when the particles’ magnetic moment can freely fluctuate and is aligned by the application of external magnetic field. When the ordering field is absent, the nanoparticles exhibit negligible net magnetization and, consequently, coercivity is zero. Value of saturation magnetization was the highest at non-ligand-coated sample **S1**, at 5 K ~ 38 emu/g, and its significant decrease was observed at ligand-coated samples (**S2**, **S3**, **S6**) as well as at porous silica shell coated samples (**S22** and **S23**). This phenomenon was expected due to the decreasing concentration of magnetic Fe_3_O_4_ amount in the volume of studied samples, see y-axis values in Fig. 11, (magnetization is calculated as total magnetic moment of the sample per its mass).

In spite of the wealth collection of *M(H)* data, the exact determination of the saturation magnetization of the iron-oxide core and subsequent estimation of the average thickness of non-magnetic shell of corresponding NP system is not straightforward. This is due to the well-known fact that magnetic properties of the Fe_3_O_4_ NPs are drastically affected by the presence of irregularities and microstrain in the lattice. As it has been documented by^41^ well crystalline Fe_3_O_4_ NPs of sizes similar to ours (~ 10 nm) may exhibit *M*_*s*_ values in the wide range from 78 emu/g (microstrain ε = 0.43 × 10^–3^) down to 41 emu/g (ε = 3.1 × 10^–3^). Since the microstrain determined for our reference sample **S1** is even higher (ε = 3.75 × 10^–3^), we can suppose that the saturation magnetization of our iron-oxide cores is rather closer to 41 emu/g than to its bulk value 83 emu/g^41^. With the aid of experimental data, we can estimate the upper limit of silica shell coating the iron-oxide beads in the sample **S1**. When taking into account *M*_*s*_(S1, 300 K) = 33.5 emu/g, average core size *D*_*XRD*_ = 11 nm and upper limit *M*_*s*_ ~ 80 emu/g for magnetite nanoparticles of density 5170 kg/m^3^, one obtains maximum thickness ~ 3 nm of non-magnetic SiO_2_ shell (density 2650 kg/m^3^).

**Figure 12:**
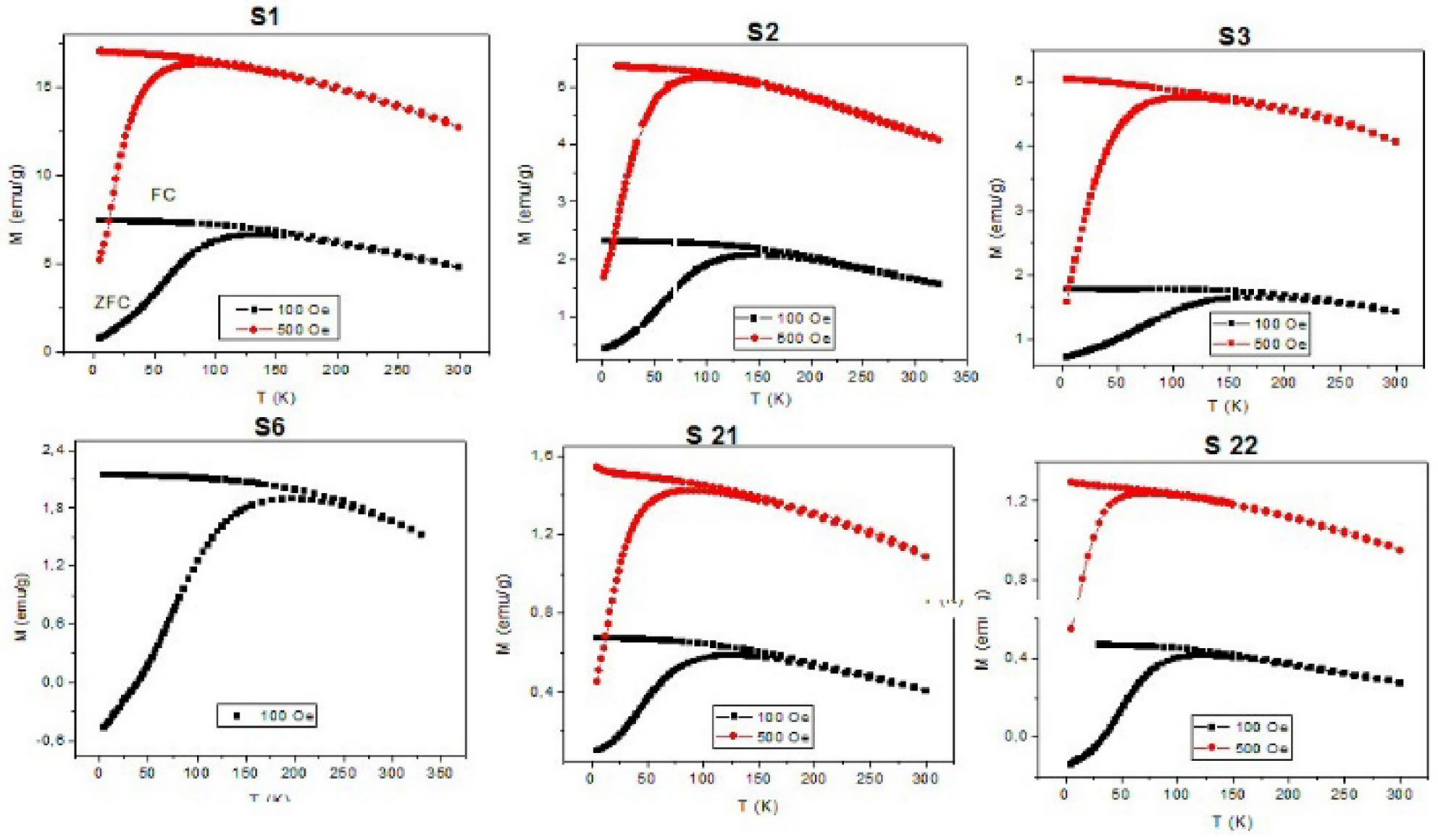
Magnetization vs temperature measured in ZFC/FC protocols for selected studied samples.

The influence of capping layer (ligands and porous silica) on the magnetic performance of the prepared systems has been investigated by means of zero-field cooling (ZFC) and field cooling (FC) magnetization vs temperature measurements, see Fig. 12.

Since from the magnetic measurements only the properties of magnetically active compounds (Fe_3_O_4_ core) can be observed, we calculated the size of magnetic core for each sample by modeling of experimental ZFC/FC magnetization data (Supplementary Information) and results are presented in Table 1.

Generally speaking, from the ZFC/FC magnetization one can derive basic characteristics of a superparamagnetic nanoparticle system. The position of the ZFC maximum is related to the blocking temperature of the system that is, in the case of the non-interacting system, determined by the activation energy barrier *E*_a_ = *K*_*eff*_*V*_*mag*_, where *K*_*eff*_ is magnetocrystalline anisotropy constant and *V*_*mag*_ magnetic volume of the particle. The presence of interparticle interaction increases the *E*_a_ value, shifting the ZFC maximum towards higher temperatures^42^. We have fitted the ZFC model to the experimental data and in the case of **S1** sample we have obtained *K*_*eff*_ = 1.8 × 10^4^ J/m^3^ what is in excellent accordance with the reference values reported for Fe_3_O_4_ NPs^41^. However, the particle diameter (median of size log-normal distribution) *D*_*mag*_ = 13 nm (σ = 0.21 nm) determined from its magnetic volume is slightly larger than *D*_*XRD*_ = 11 nm. This effect can be explained by the interparticle interactions that increase the energy barrier *E*_a_ = *K*_*eff*_*V*_*mag*_ and therefor the apparent volume of the NPs appears larger. The presence of interparticle interaction is also evident from the FC curve that exhibits temperature independence (plateau) at low temperatures instead of superparamagnetic increase typical of non-interacting systems^43^. We have modelled the FC curve by employing the *K*_*eff*_ and *V*_*mag*_ values obtained from ZFC fit, Fig. 16, however, the discrepancy between the model and experimental data is apparent. When we fitted the model to the experimental FC data (Fig. 16), we obtained very similar size distribution (*D*_*mag*_ = 13 nm, σ = 0.25 nm) when compared to ZFC data, but *K*_*eff*_ = 4.5 × 10^4^ J/m^3^ has been found significantly larger. The effective value of magnetocrystalline constant is larger than expected for non-interacting Fe_3_O_4_ NPs and its apparent enhancement can be ascribed to the effect of interparticle interactions. The presence of interparticle interactions in the system has been evidenced also by other methods (Figs. C5 and C6 in Supplementary Information).

The ZFC/FC data can also be used as a complementary data for the evaluation of capping layer (organic ligand, porous silica) formation and its distribution. From the mutual comparison of the curves, Fig. 12, one can see that that ZFC maximum is located at T ~ 150 K and does not shift significantly within the sample series. On the other hand, the magnitude of magnetization decreases varies consistently as it is in the case of isothermal magnetization data, Fig. 11, Table 1. The experimental data can be explained by the scenario, that the capping material is covering rather the aggregates of nanoparticles than individual particles. The presence of non-magnetic material among the NPs would increase the interparticle distances and consequently would decrease the strength of interactions. That would be manifested both by the shift of the ZFC maximum towards lower temperatures and by the increase of FC magnetization when approaching lower temperatures. The hypothesis on aggregate formation is also supported by the TEM data (Supplementary Information). Virtually, the iron-oxide cores have been found in groups, where their mutual distances are similar as those evident form the TEM micrograph corresponding to **S1**, Fig. 4.

### Application of eMNPs for isolation of viral RNA from tissue sample

**Figure 13:**
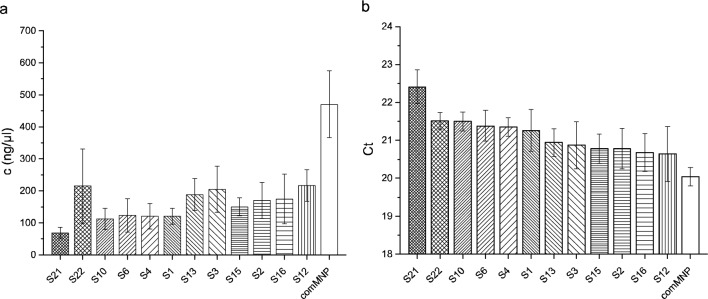
Testing of eMNPs samples for isolation of RNA and in RT-qPCR assay for the detection of HEV from pig liver tissue. (**a**) Concentrations of isolated RNA, (**b**) C_t_ values of RT-qPCR (lower C_t_ value indicates earlier PCR amplification of the target due to the higher concentration of isolated RNA in the sample). The values represent average of five repeated analyses.

Initially, the whole collection of the prepared eMNPs was applied for isolation of viral RNA from pig liver infected by HEV. Our experiments demonstrate that all eMNPs tested were efficient for isolation of viral RNA from liver tissue of infected pig and could be used in RT-qPCR. No doubt, the concentration of isolated RNA (Fig. 13a) was the highest with comMNPs (470 ng/µL). Of eMNPs tested, the highest value was obtained with sample **S12** (217 ng/µL), the lowest with sample **S21** (69 ng/µL). The purity of RNA isolated by comMNPs and by eMNPs was comparable varying in the range A260/280 = 1.97–2.10.

When evaluating the quality of eMNPs by C_t_ values of RT-qPCR (Fig. 13b), which is the main parameter in diagnostic laboratory, the encouraging results were obtained. While C_t_ with commercial magnetic beads varied at value 20.0, the **S1** sample with no ligand bound on magnetic core provided average C_t_ = 21.2 which indicates just slightly less efficiency of virus detection. However, the samples **S12** and **S16** as representatives of magnetic bead coated with functional ligands of modification A (see Fig. 13) provided average C_t_ = 20.6, which is comparable to the beads from commercial kit. On the other hand, for samples **S21** and **S22** as representatives of modification C resulted in the average C_t_ = 22.4 and 21.5, respectively.

The inspection of chemical character of ligands bound to magnetic core revealed that the best detection of HEV close to commercial kit provided the ligands **MMSP** (sample **S12**) and **AEAPTMS** (sample **S16**), Fig. 13. Other ligands under **strategy A** (samples **S2**–**S16)** and **strategy B** (samples **S21** and **S22**) were of less efficiency.

### Application of eMNPs for isolation of viral RNA from biofluid samples

Six selected eMNPs (**S1**,** S3**,** S6**,** S21**, S**22** and **S23**) were further used to isolate viral RNA from biofluid samples (nasopharyngeal swab samples of patients infected with SARS-CoV-2). The selected eMNPs represent all three groups of the prepared eMNPs, magnetic nanoparticles without ligands or surface modification (sample **S1**), ligand-coated eMNPs samples (samples **S3** and **S6**), and porous silica shell coated eMNPs samples (samples **S21**, **S22**, and **S23**).

**Figure 14:**
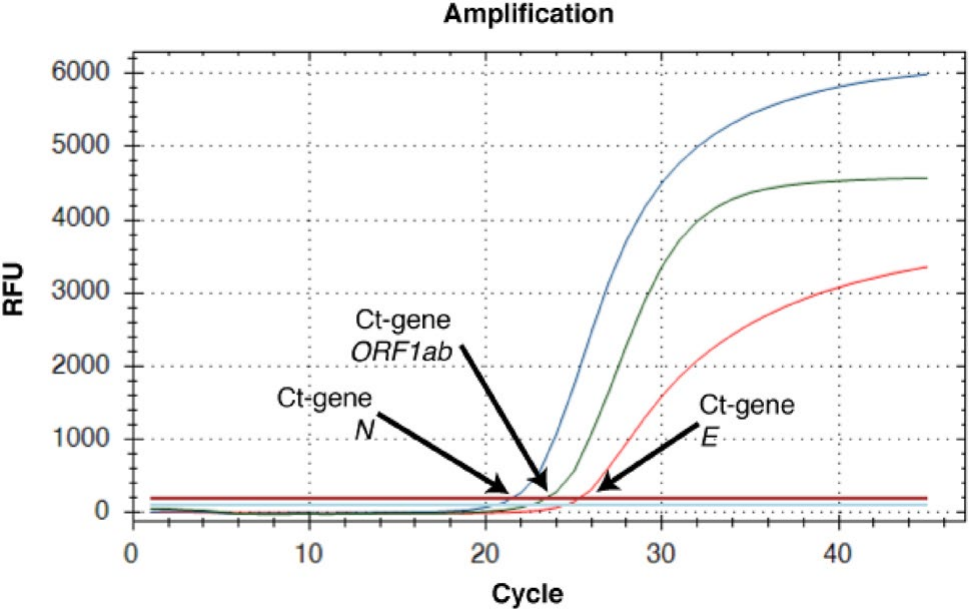
Typical RT-qPCR detection of SARS-CoV-2 RNA isolated from swab samples of Covid-19 positive patients. SARS-CoV-2 specific genes were detected by COVID-19 Real Time Multiplex RT-PCR Kit (Labsystems Diagnostics) at channels: FAM (*E* gene, blue line), HEX (*ORF1ab*, green line), TEXAS RED (*N* gene, red line) and CY5 (IC, violet line).

Although the concentration of isolated RNA from clinical samples was under limit for reliable spectrophotometric measure at A260/280 nm, isolation of viral RNA from the biofluid/swab samples was confirmed by RT-qPCR. The acquired C_t_ values of the detected viral genes indicate that the prepared eMNPs bound virus RNA in very high rate and have high potential for RNA isolations. The viral RNA (all three simultaneously analyzed SARS-CoV-2 genes—*ORF1ab*, *E* and *N* gene) was detectable in all tested isolates acquired with each sample of eMNPs, it means that application of any prepared eMNPs in viral RNA isolation and subsequent detection of coronavirus would prove SARS-CoV-2 infection in each analyzed clinical sample. Individual viral genes in an isolate were detected at different C_t_ values, as shown at Fig. 14. Obviously, the C_t_ values of the detected viral genes in the RNA isolates varied also depending on the clinical sample and the viral load present in the sample.

**Figure 15:**
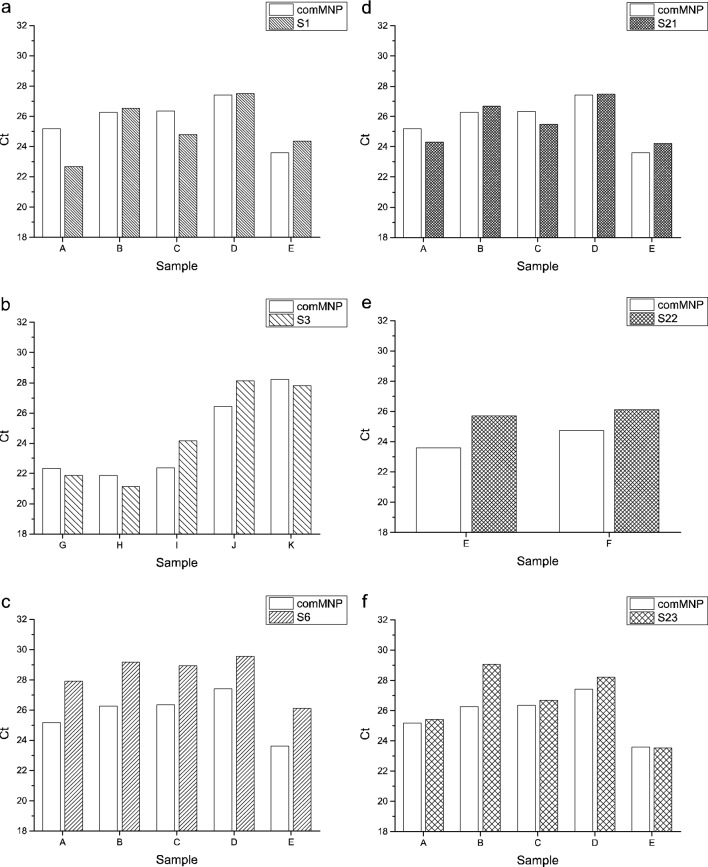
The RT-qPCR detection of SARS-CoV-2 RNA isolated by different eMNPs. Comparison of C_t_ values of SARS-CoV-2 *ORF1ab* gene fragment detected in RNA isolated from clinical swab samples of COVID-19 affected patients using commercial (comMNPs) and experimental magnetic nanoparticles (eMNPs)—**S1**, **S3**, **S6**, **S21**, **S22** and **S23**.

The virus RNA isolated by comMNPs (here scored by C_t_ values of *ORF1ab* gene amplicon) was detected in the analyzed samples between C_t_ = 21.9 to 28.2, see Fig. 14. According to the C_t_ values, similar amounts of viral RNA was also acquired with eMNPs without ligand layer (sample **S1**) and porous silica shell coated eMNPs sample (sample **S21**, Fig. 15a,d). In comparison to the comMNPs, slightly lower isolation efficiency was shown by another two surface-modified eMNPs (sample **S3** and **S23,** Fig. 15b,f). The lowest efficiency of RNA isolation in the used kit lysis buffer was acquired with surface-modified eMNPs—sample **S22** and **S6** (Fig. 15e,c), with evidently higher C_t_ values of detected *ORF1ab* gene in comparison to comMNPs isolates. Similar results were obtained also if the other two detected viral genes (*E* and *N*) were taken in account (data not shown). As expected, the prepared eMNPs bound whole pool of RNAs present in the experimental sample, i.e. the viral RNA as well as human RNAs in the swab sample. Detection of the co-purified human RNA represented by selected human cellular mRNA (IC control) was used to monitor correct sampling and successful isolation and RT-qPCR procedure. The ratio between viral RNA and the IC was stable for each biological sample analyzed, regardless of the eMNPs used for the RNA isolation (data not shown).

## Discussion

In our work we prepared broad series of eMNPs with three different strategies as indicated in Fig. 1. The three strategies have been selected in order to examine fundamentally different capping layer composition, structure and organization with respect to efficiency of virus isolation. The application of the prepared eMNPs for isolation of viral RNA was tested on two different kinds of clinical samples and viruses in diagnostic RT-qPCR. One sample represented the complex biological sample—tissue from liver pig naturally infected with hepatitis E virus—HEV, while the others were biofluid samples—swab samples from patients infected by SARS-CoV-2.

### Structural and magnetic characterization

All of the studied samples (with the exception of sample **S23**) are based on the same core structure that is composed of magnetic Fe_3_O_4_ NPs coated by amorphous silica shell (sample **S1**). The magnetic properties of the modified samples **S2–S22** are therefore determined by the magnetic behavior of the sample **S1**. We have studied this sample in more details in order to use the data as a reference for the evaluation of the rest of the series of modified samples.

**Figure 16:**
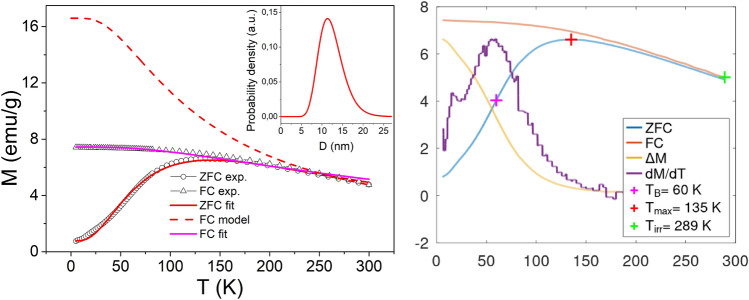
Analysis of magnetic properties of the sample **S1** by the ZFC/FC model (left panel). The model of ZFC (red curve) and FC (magenta curve) magnetization are fitted to the experimental data (empty symbols). Red dashed curve represents the model of FC curve in the case of non-interacting system. The particle magnetic size distribution obtained from the model fitting is shown in the inset. Right panel: Relevant temperatures characteristic of the examined system. The blocking temperature (*T*_*B*_) is determined as the maximum of derivative *dM/dT* of the difference Δ*M* = FC − ZFC. *T*_*max*_ is the temperature of ZFC maximum and *T*_*irr*_ the irreversibility temperature where ZFC and FC curves separate.

The analysis of X-ray diffraction pattern of the sample **S1** (Fig. 6, Fig. C3 in Supplementary Information) along with XPS (Fig. 8) analysis confirmed the presence of well crystallized pure Fe_3_O_4_ phase without evidence of other significant crystalline phases. The average crystalline domain size that corresponds to the typical Fe_3_O_4_ core of the particle has been determined to *D*_*XRD*_ = 11 nm. This is consistent with the average total size of the particle observed by SEM *D*_*SEM*_ = 20 nm, Fig. 7. If we assume spherical shape of the particle, we obtain the average thickness of amorphous silica shell (*D*_*SEM*_–*D*_*XRD*_)/2 ~ 5 nm. The average total size of the particle observed by TEM *D*_*TEM*_ = 9.5 nm is slightly smaller than expected based on XRD and SEM data. We attribute the discrepancy to the accidental selection of a region with population of “smaller” NPs during TEM experiments. Since the size distribution estimated from the micrographs taken by electron microscopy (TEM/SEM) provides information only from a local region, it is possible that it is not fully representative of the whole sample. On the other hand, X-ray measurements are performed on the bulk sample and hence provide information on the crystallite size averaged from a large sample volume (with respect to TEM/SEM). Thus, we consider *D*_*TEM*_ as the plausible average Fe_3_O_4_ core size. In fact, this value has been found in good accordance with the magnetic core size *D*_*mag*_ established from the analysis of magnetic measurements. Likewise, in the case of XRD, magnetic measurements are performed on the bulk sample (typically few miligrams) and therefore the magnetic properties are averaged through the whole volume of the examined system. The magnetic core size has been established employing fit of the theoretical model^44^ (Supplementary Information: Zero field cooling (ZFC) field cooling (FC) magnetization modeling) to the experimental ZFC–FC *M(T)* data of the sample **S1**. The model assumes (i) superparamagnetic system, (ii) no interactions, (iii) linear field response, (iv) uniaxial particle anisotropy, (v) lognormal particle volume distribution. The best model fit, Fig. 16, provided median magnetic core diameter *D*_*mag*_ ~ 13 nm. Although the value is slightly larger than *D*_*XRD*_, it reconfirms the hypothesis that the presented TEM size distribution is not plausibly representative of the whole sample. In fact, finding the magnetic core diameter larger than average Fe_3_O_4_ crystallite size is not a striking result.

This is the effect of strong interparticle interactions evidenced in all of the examined samples. The signature of strong interactions can be easily recognized by temperature independence of FC magnetization below system’s blocking temperature (usually associated with the maximum of ZFC curve), Fig. 16. In order to demonstrate the effect of interactions, we have modeled the FC curve (red dashed curve) employing the parameters obtained from the fit to the ZFC data (red curve) of corresponding **S1** system, Fig. 16. One can clearly recognize the large discrepancy between the FC model and experimental FC data (empty triangles). This is attributed to the effect of strong interparticle interactions. However, the presence of interactions in the nanoparticle system increases the energy barrier *E*_*a*_ that particle’s superspin has to overcome to flip its orientation. The larger energy barrier is manifested by the shift of the ZFC maximum to higher temperatures with respect to the system of the same, but non-interacting NPs. Since *E*_*a*_ is directly proportional to the magnetic volume of the particle, from the ZFC model fit we obtain larger effective volume of the particle than physical volume of the crystallite. Virtually, the blocking temperatures values of non-interacting Fe_3_O_4_ NPs with sizes comparable to our *D*_*XRD*_ = 11 nm are reported to be lower^45^.

Similar to our preparation **strategy A**, the modification of the MNPs with different ligands bound to surface of magnetic core were used for isolation of virus nucleic acid and subsequent detection of virus by PCR for RNA viruses^12^ including SARS-CoV-2^22–24^ and DNA viruses^21,25^. The successful modification of Fe_3_O_4_@SiO_2_ beads by organic ligands (**Strategy A**) has been evidenced both by FTIR spectroscopy (Fig. 9) and by the SEM, Fig. 7. The comparison of SEM size distributions of ligand-modified samples with the reference sample **S1** suggests the presence of organic shell of large thickness (above 20 nm, Fig. 5, Table 1) or the presence of the aggregates of NPs covered by the ligand layer. The hypothesis is based on the fact that organic ligand would produce monolayer of the thickness roughly corresponding to the size of the molecules (e.g. ~ 1 nm for APTES)^46^ employed for its creation. The actual thickness of the specific ligand layer is difficult to estimate even with the aid of the data from TEM or XRD, since organic compounds are not visible employing the two methods. Here we can turn to the magnetometry data that provide the support for the formation of thicker function layer on the beads’ surface. One can clearly observe a significant decrease of magnetic characteristics (*M*_*s*_, Table 1, Fig. 11) when compared the modified samples with the reference **S1** sample. According to the XRD data, the magnetic Fe_3_O_4_ cores have not been affected during the modification process (phase composition, size). Therefor observed reduction of the *M*_*s*_ (sample’s magnetic moment per its mass) up to 3 times could be plausibly explained by the presence of significant amount of organic compound in the sample. Besides of this, it is very difficult to recognize the boundaries of the individual particles from SEM micrographs. Therefore we cannot rule out the possibility that the objects we observe are in fact the agglomerates of individual particles, similar that has been reported elsewhere^47^. The hypothesis on agglomerates is supported also by magnetization data. The presence of intermediate interparticle magnetic interactions has been evidenced in all of the examined powder samples by several methods (see Figs. C5 and C6 in Supplementary Information). In the case of thick non-magnetic shell as estimated from SEM (Table 1) a typical particle center-to-center distance would be larger than 60 nm. For the magnetic cores of sizes similar to ours this would result in a significant suppression of mutual interactions^48^ and we should observe superparamagnetic behavior as demonstrated by the model of FC curve in the Fig. 16. However, as it is apparent from Fig. 12, this is not true even for the samples with the largest SEM sizes.

In the case of nanoparticle modification according to the **Strategy B**, it was necessary to evidence the presence of mesopores in the outer silica shell. For this purpose, nitrogen adsorption/desorption measurements have been performed, Fig. 10. The data clearly suggest the organization of mesoporous structure on the nanoparticle surface. Owing to the pores, the specific surface area *S*_*BET*_ of amorphous silica has increased up to the order of magnitude (**S21** vs. **S1**) being comparable with the regular mesoporous structures SBA-15 or SBA-16^49,50^. The potential benefit from this modification is straightforward. Enlarged surface provides more silane groups that are available for binding the RNA what is improving the efficiency of the particles. On the other hand, magnetization data (Figs. 11 and 12) show significant reduction of magnetic properties of the systems modified by porous silica shell (**S21, S23**). The magnetization (magnetic moment of the sample per unit mass) decreased over an order of magnitude when compared to untreated beads (**S1**). This suggests formation of a rather thick outer silica layer on the surface of **S21** and **S22.** If we assume a simple spherical core-multishell model for a typical particle, the layer would correspond to approximately 16 nm thick homogenous SiO_2_ shell of the density 2.65 g/cm^3^. However, due to the presence of the void pores, the average density is significantly reduced and the thickness of the porous layer extends accordingly beyond the 16 nm.

Lastly, let us turn to the modification **Strategy C**. The MNPs prepared under these conditions exhibit unique shell structure consisting of organic and inorganic layers that are in inverted order as usually, Fig. 3. The aim was to propose and examine the efficiency of different approach in the magnetic core surface modification. Despite the lower value of the internal surface area in the sample **S23** of *S*_*BET*_ ~ 170 m^2^/g (with respect to the **S21** and **S22** samples) the sample **S23** shows a comparable efficiency for isolation of RNA from biofluid/swab, see Figs. 14 and 15.

Large absolute values of Zeta potential (ZP) (e.g. samples modified by silica **S1**, **S21**, **S22**) indicate that there is electric charge concentrated on the surface of our NPs systems. The charge facilitates mutual repulsion of the particles and, consequently, the stability of their dispersion in water with neutral pH. However, even for the systems exhibiting the highest magnitudes of ZP, the experiments employing dynamic light scattering (not presented in this study) show on average hydrodynamic size of the objects larger than ~ 150 nm. Along with the evidence on large polydispersity, we can conclude on the presence of stabile dispersion of nanoparticle agglomerates rather than individual particles. Since our nanoparticle systems are designed for “in vitro” testing, the presence of agglomerates does not hamper their effective application. Moreover, the advantage that we are not strictly constrained to physiological conditions allows for significant tuning of ZP of our systems. This can be easily done either by changing the pH of the dispersing medium, or by the modification of the particle surface by the ligands containing both acidic (thiol, MPTMS, **S3**) and basic (amines, APTES, **S2**) functional groups^51^. By changing the polarity of nanoparticles’ ZP, one can target and electrostatically attract specific (complementary) segments of RNA, while by increasing the magnitude of ZP the segment capture efficiency can be improved. Hence, there is still a range of methods for tailoring and further advanced modification of the presented nanoparticle systems regarding their application potential.

### Prepared eMNPs bind efficiently viral RNA

We proved efficient reversible adsorption of RNA to the prepared eMNPs during isolation of viral RNA using commercially available nanobeads-based RNA isolation kits, in which the original/commercial magnetic beads (comMNPs) were replaced by our experimental MNPs (eMNPs). We showed that the eMNPs-isolated viral RNA is readily detectable by RT-qPCR with a comparable sensitivity to detection of viral RNA isolated by comMNPs.

The testing of 12 eMNPs on pig liver sample demonstrated that RNA was successfully isolated independently if they were prepared by any of three synthetic strategies despite tissue contained broad spectrum of interfering agents and RNase activity. This RNA could be used in diagnostic RT-qPCR in which C_t_ values were comparable or close to those obtained by commercial kit. The ligands bound to magnetic beads influenced the binding of RNA not more than one order.

Similarly high efficiency of viral RNA isolation by the tested eMNPs was acquired also with biofluid/swab samples infected with SARS-CoV-2 virus. With all tested eMNPs we were able to isolate high quality viral RNA. The comparison with commercial MNPs shows comparable yield of isolated viral RNA. It turned out that two samples unmodified **S1** and porous surface modified **S21** MNPs exhibit the results of efficiency similar to comMNPs.

Although we expected that the surface modification and surface bound ligands could provide higher capacity for binding RNA and therefore higher effectivity of RNA isolation we did not observe significant improvement in the isolation process with the commercial kits used for RNA isolation. The reason could be saturation of the isolation process, weaker interaction of the modified surface with RNA in the lysis/binding solution or contrariwise too strong affinity of the ligand groups with RNA and problems with elution/desorption efficiency. Our initial RNA isolation trials clearly and directly show that the prepared eMNP are capable to absorb nucleic acids (NA) on its surface in complex clinical samples. For better understanding of the NA/eMNP interaction in the extraction solution adsorption/desorption kinetic study in precisely controlled extraction condition would be needed. However, we should point out that using the IVD certified SARS-CoV-2 kit, we always detected all tested SARS-CoV-2 genes in the Covid-19 positive clinical samples, which unambiguously confirmed that RNA isolated using our eMNPs is suitable for clinical diagnostics.

We have not tested what kind of absorption was between viral RNA and silane MNP. However, taken data from literature absorption can be result of electrostatic interaction between negative phosphate groups of RNA and positive charged amino groups on surface of MNP. Alternatively, hydrophobic interaction between MNP and RNA molecules can also take place^53^.

Thus, the prepared eMNPs appear to be very suitable for the isolation of viral RNA, or cellular RNA from biofluid samples as well as from animal tissues. We should point out that eMNPs were tested using screening approach, just simple replace of magnetic beads in commercial kit with our eMNPs. The observed differences in the efficiency of RNA isolation using individual MNPs in the used models probably reflect the different environment/conditions for the adsorption of RNA to eMNPs in the lysis/binding buffer, which significantly affect the interaction of RNA and MNPs^18^. The isolation efficiency can be influenced e.g. by the concentration of guanidine thiocyanate, DTT, pH of the lysis buffer^52^ or the character of ligands bound to magnetic core and size of magnetic beads.

## Conclusion

Our work is one of the most extensive studies of functionalized MNPs, which has greatly expanded the knowledge on the preparation of high-quality magnetic particles for the isolation of nucleic acids from biological samples with applications in the diagnostic practice of infectious diseases in humans and animals. Several strategies, including organic ligand or an inorganic layer capping, were designed and tested in order to improve the MNP performance. The work demonstrated that some of our proposed MNPs exhibited properties comparable to magnetic beads used in commercial kits dedicated for molecular diagnostics of viral infections. These positive results were acquired by only simple replacement of commercial MNPs by our modified experimental MNPs in commercial isolation kit without more extensive optimization of the isolation procedure. Apart from this, there is still a wide range of possibilities for further tuning the efficiency of our MNP systems. As it is discussed, this can be done either by mixing the specific ligand species on the particle surface. Or, in addition, in the future we would like to optimize the conditions for the extraction and binding of RNA to MNPs during RNA isolation by adjusting the pH according to the measured potential of the MNPs in solution. Therefor we can conclude that systematic synthesis of magnetic particles with different ligands bound to the magnetic core is perspective approach to find particles with better properties for application in biological research, diagnostics and therapy. We believe that further optimization of all steps in workflow synthesis and analysis will lead to more sensitive diagnostic assays which significantly improve our struggle not only with COVID-19 pandemics but also with other viral epidemics in future.

### Supplementary Information

Supplementary Information.

## Data Availability

Our analyzed datasets are available from the corresponding author on reasonable request.
