# Supplementary material for: The surface modification of the silica-coated magnetic nanoparticles and their application in molecular diagnostics of virus infection
Source: Sci Rep. 2024 Jun 23;14:14427. doi: 10.1038/s41598-024-64839-2 (PMC11194262; doi:10.1038/s41598-024-64839-2)
Supplement: Supplementary file 1 — Supplementary Information. [file 41598_2024_64839_MOESM1_ESM.docx]

**SUPPLEMENTARY INFORMATION**

**The surface modification of the silica-coated magnetic nanoparticles and their application in molecular diagnostics of virus infection**

A. Zeleňáková^a^*, V. Zeleňák^b^, E. Beňová^b^, B. Kočíková^c^, N. Király^b^, P. Hrubovčák^a^, J. Szücsová^a^, L. Nagy^a^, M. Klementova^d^, J. Mačák^e^, V. Závišová^f^, J. Bednarčík^a^, J. Kupčík^d^, A. Jacková^c^, D. Volavka ^a^, J. Košuth^g^ , Š. Vilček^c^

*^a^Institute of Physics, Faculty of Science, P.J. Šafárik University, Park Angelinum 9, 04001 Košice, Slovakia*

*^b^Institute of Chemistry, Faculty of Science, P.J. Šafárik University, Moyzesova 11, 04001 Košice, Slovakia*

*^c^Department of Epizootiology, Parasitology and Public Health Protection, University of Veterinary Medicine and Pharmacy in Košice, Komenského 73, 041 81 Košice, Slovakia*

*^d^Institute of Physics of the CAS, v.v.i., Na Slovance 1999/2, CZ-182 21 Praha 8, Czech Republic*

*^e^synlab slovakia s. r. o Department of clinical microbiology, Opatovská cesta 10, 04001 Košice, Slovakia*

*^f^Institute of Experimental Physics, Slovak Academy of Sciences, Watsonova 47, 04001 Košice, Slovakia*

*^g^Institute of Biology and Ecology, Faculty of Science, P.J. Šafárik University, Šrobárova 2, 04154 Košice, Slovakia*

*Corresponding author: adriana.zelenakova@upjs.sk

**TEM size determination and phase composition confirmation**

The frequency histograms of nanoparticle iron-oxide core sizes have been obtained by taking into account at least 100 nanoparticles from the TEM micrographs. The Gauss and Log-normal functions have been fitted to the obtained size distributions. Since slightly better fits have been obtained by employing Gauss function the values reported in our study correspond to Gauss fit, see Fig. C1. In order to confirm that no oxidation of the magnetic core occurs during surface modification using **strategies A, B and C**, a comparison of electron diffractograms documenting the presence of a pure magnetite phase in all of the samples is shown, Fig. C2.


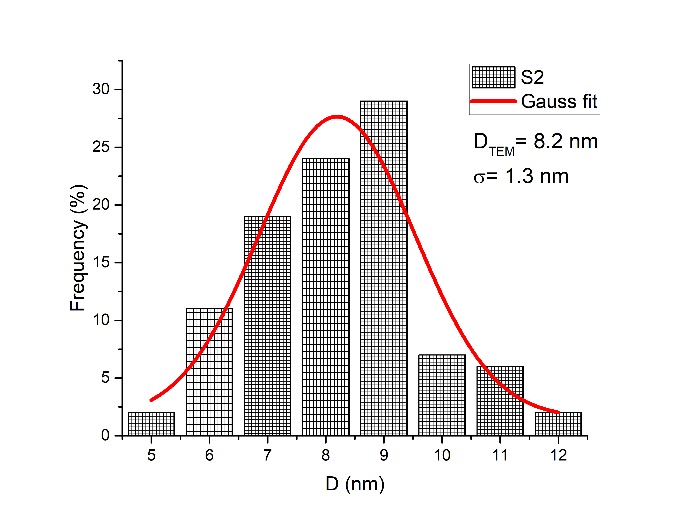

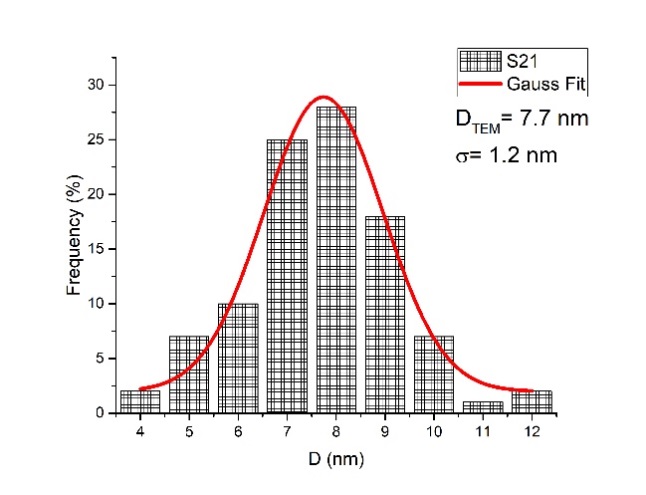


Supplementary Fig. C1: Iron-oxide core size distribution based on TEM experimental data for the samples **S2 (**represents **Strategy A)** and **S21 (**represents **strategy B)**. Red lines represent Gauss function fits to the experimental data.


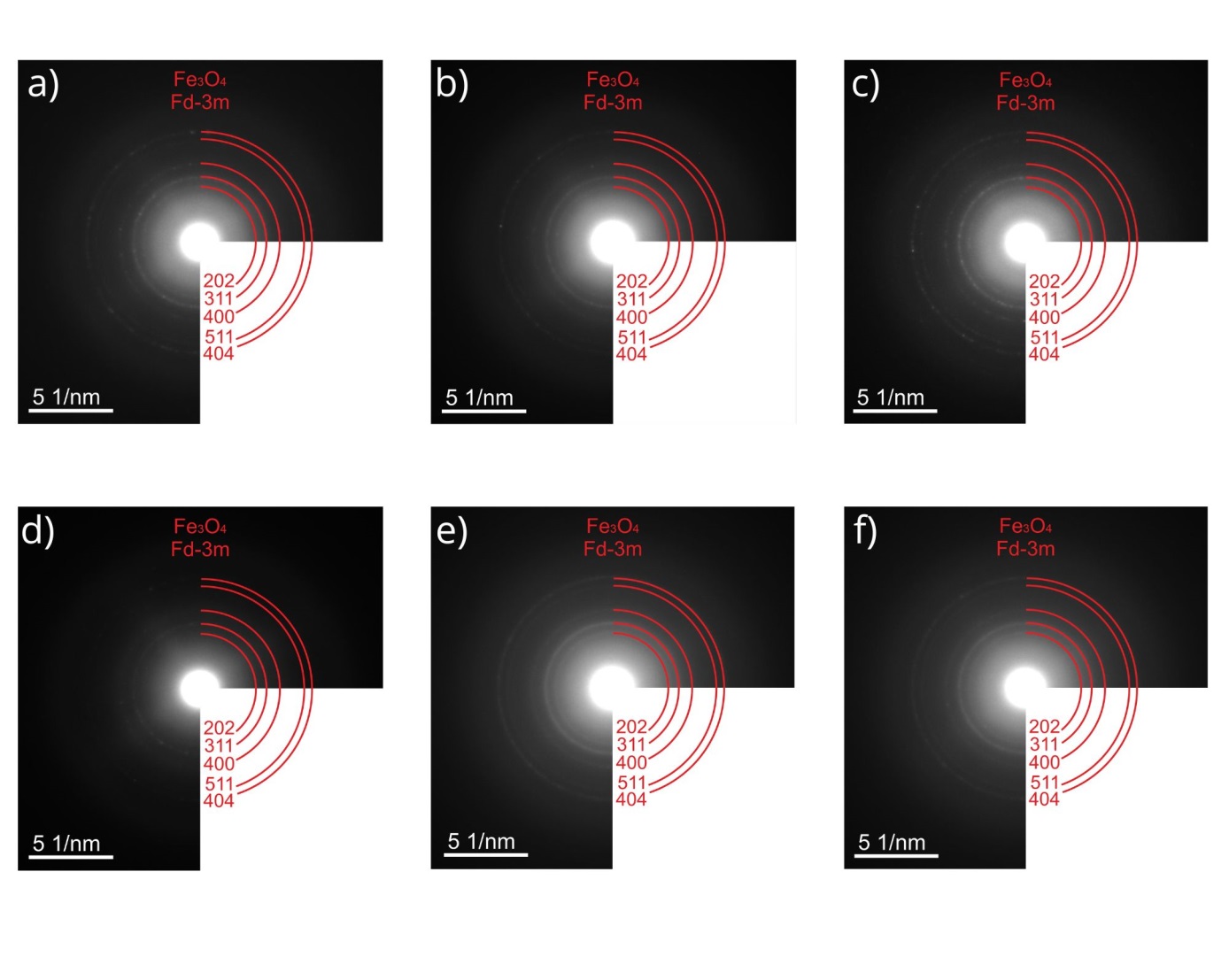


Supplementary Fig. C2: Electron diffraction pattern from TEM measurements of samples prepared by **strategies A, B, and C** where the same magnetic core **S1** is present. a) Sample **S2**, b) sample **S3**, c) sample **S4**, d) sample **S6**, e) sample **S21**, f) sample **S23**.

**XRD size determination**

The X-ray diffraction profile was modeled using the Lorentzian function. A standard LaB_6_ sample was measured to determine the background resolution function of the instrument. The crystallite size along with microstrain ε in the magnetic beads (**S1** sample) have been determined by Williamson-Hall method^1^, where the parameters are obtained from the linear fit of the plot βcosθ = Kλ/D_XRD_ + 4εsinθ. In the equation, K = 0.94 is shape factor corresponding to the nanoparticles with cubic symmetry, λ = 0.154 nm is the X-ray wavelength, β is full width at half maximum of corresponding peak and θ its position.

The rest of the sample series (surface modified samples with the **S1** core) were analyzed using the Scherrer equation^2^ where average crystallite size is calculated as *D_XRD_* = Kλ/βcosθ. The Scherrer equation has been applied to the peak exhibiting the highest intensity (311) from the diffraction pattern of corresponding sample.


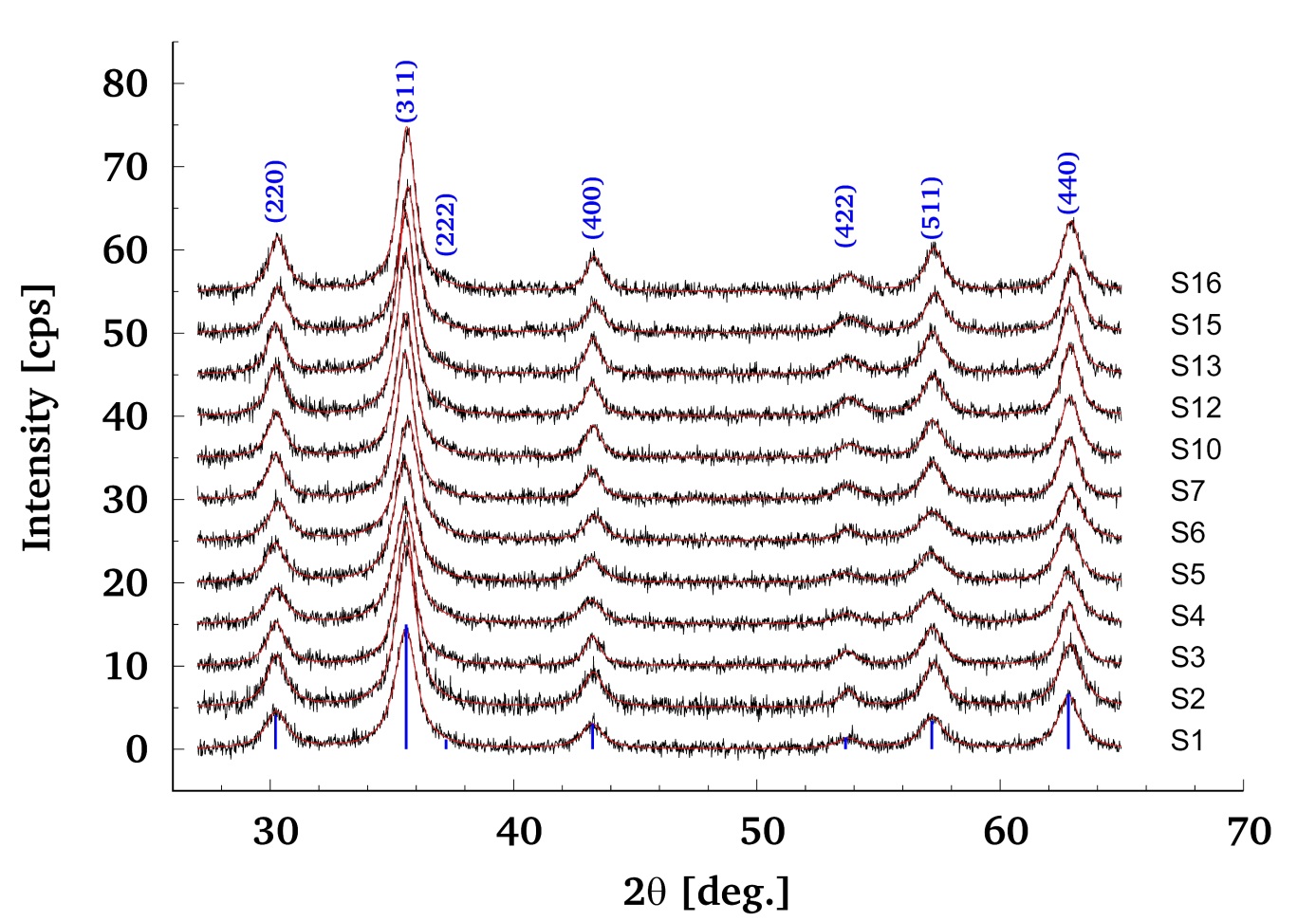


Supplementary Fig. C3: Comparison of X-ray diffraction patterns of samples **S2 - S16** with organic ligand surface layer with non-coated sample **S1** confirming the presence of cubic Fe_3_O_4_ phase (space group 227, Fd-3m) and the best model (red line) that fits the experimental data of the examined sample series. Blue lines marked the amplitudes and the position of the Bragg peaks associated with Fe_3_O_4_ cubic phase.


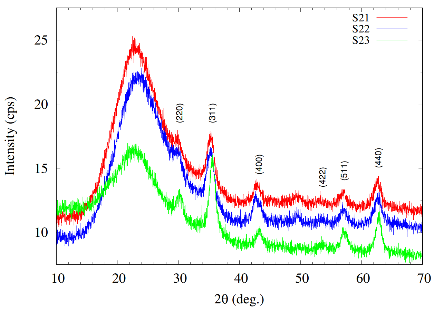


Supplementary Fig. C4: XRD pattern of the samples **S21, S22, S23** modified by the porous silica shell with the same core as modified samples **S2 - S16** confirming the presence of cubic Fe_3_O_4_ (space group 227, Fd-3m).

**Zero field cooling (ZFC) field cooling (FC) magnetization modeling**

The ZFC/FC magnetization experimental data have been fitted by the semi-analytical model that has been introduced by Tamion et. al.^3,4^. The model assumes no interparticle interactions and therefor switching field frequency dependence on temperature is defined

$\nu\left( T \right)=\nu_{0}exp\left[ \frac{-K_{eff}V_{mag}}{k_{B}T} \right]$, (S1)

where ν_0_=10^9^ Hz is the attempt frequency. The temperature sweeping rate during the experiment (2 K/min) is reflected in the characteristic time δ_t_(T) and the system’s magnetic moment can be expressed as

$m_{ZFC}\left( T \right)=N_{T}\int_{0}^{\infty} M_{0}V_{mag}\left[ e^{-{\nu\left( T \right)\delta}_{t}(T)}+ \frac{K_{eff}V_{mag}}{k_{B}T}\left( 1-e^{-{\nu\left( T \right)\delta}_{t}\left( T \right)} \right) \right]P\left( D_{mag} \right)d\left( D_{mag} \right)$,

$m_{FC}\left( T \right)=N_{T}\int_{0}^{\infty} M_{0}^{'}V_{mag}e^{-{\nu\left( T \right)\delta}_{t}(T)}+ \frac{\mu_{0}m_{s}^{2}H}{{3k}_{B}T}\left( 1-e^{-{\nu\left( T \right)\delta}_{t}\left( T \right)} \right)P\left( D_{mag} \right)d\left( D_{mag} \right)$, (S2)

where *M_0_V_mag_=µ_0_m_s_^2^H/(3K_eff_V_mag_)* is the initial susceptibility of the system at low temperature, *P(D_mag_)* is log-normal size distribution of magnetic core diameter (estimated from TEM experimental data), *k_B_* and *µ_0_* denote the Boltzmann constant and magnetic permeability of vacuum, *H* is the applied magnetic field, *m_S_* is the saturation magnetization of the iron oxide core and *N_T_* is the number of magnetically active clusters. In the case of the FC model, *M’_0_* = *m_FC_(T→0)/N_T_V_mag_*, is graphically determined. In the fitting process, *K_eff_* and particle size distribution have been assumed as free parameters.

**The strength of the magnetic interparticle interactions**

The presence of strong interparticle interactions in the powder sample **S1** has been evidenced by several methods. The magnetization measurements performed according to specific protocols^5^ – direct current demagnetization (DCD), isothermal remanent magnetization (IRM) can be utilized for the Henkel plot construction, Fig. C5. Here, when plotted normalized values of the DCD vs. IRM, one can observe deviation of the plot from the straight line (non-interacting system). This is the signature of the presence of magnetic dipole-dipole interactions in the system^6^.


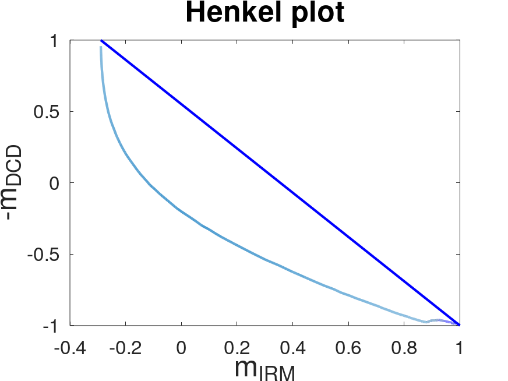


Supplementary Fig. C5: Henkel plot confirming the presence of strong dipolar magnetic interactions in the sample **S1** - magnetic beads employed for the further surface modification (**S2 - S23**).

Further evidence of the intermediate/strong interparticle interactions in the **S1** system is delivered by the analysis of the AC magnetic susceptibility (χ) vs temperature data. The three models (Neél-Arrhenius, Vogel-Fulcher and The critical slowing down)^6^ have been fitted to the maxima of the in-phase χ(T) data collected for different AC driving frequencies. The models refer to the magnetic nanoparticle systems with none, intermediate and strong interparticle interactions, respectively. The best fit to the experimental data, Fig. C6, has been obtained for the Vogel-Fulcher model hence indicating the presence of intermediate interparticle interactions in the system. The attempt frequency f_0_ has been found beyond the acceptable physical limits both for the Neél-Arrhenius (f_0_ ~ 10^19^ Hz) and for the critical slowing down model (f_0_ ~ 10^6^ Hz). The temperature *T_0_*~103 K is the parameter that refers to the increase of the energy barrier in the consequence of the intermediate interparticle interactions.


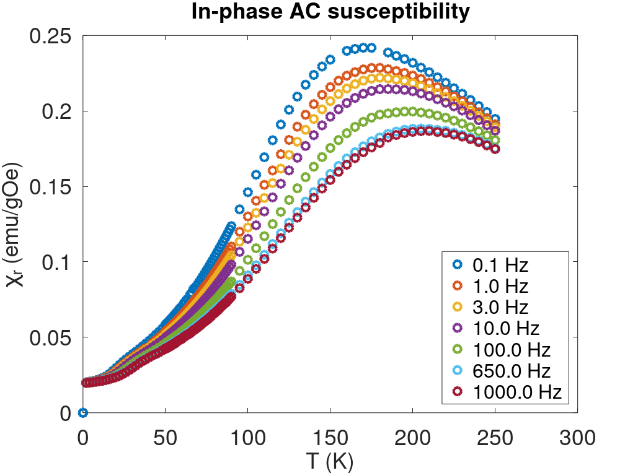

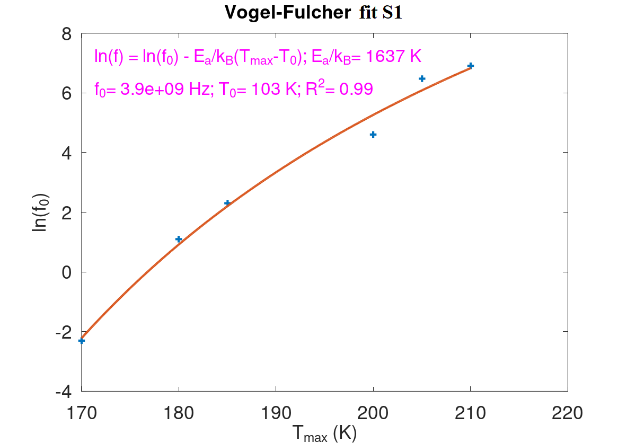


Supplementary Fig. C6: AC magnetic susceptibility data for the system **S1** and the best model fit (Vogel-Fulcher) to the maxima of the data vs temperature dependence.

Another confirmation of interparticle interactions in the **S1** system is provided by the modelling of ZFC/FC magnetization vs temperature data employing the model described above. The corresponding analysis has been performed also for the modified samples. In all of the examined samples, (i) the temperature independence of FC magnetization at low temperatures along with (ii) the shift of ZFC maximum towards higher temperatures (with respect to non-interacting Fe_3_O_4_ NPs of similar size) and (iii) significant increase of effective magnetocrystalline anisotropy constant obtained from FC fit vs ZFC fit suggest the presence of intermediate/strong interparticle interactions.

**References:**

1. Himabindu, B., Latha Devi, N. S. M. P. & Rajini Kanth, B. Microstructural parameters from X-ray peak profile analysis by Williamson-Hall models; A review. *Mater. Today Proc.* **47**, 4891–4896 (2021).

2. Patterson, A. L. The Scherrer Formula for X-Ray Particle Size Determination. *Phys. Rev.* **56**, 978–982 (1939).

3. Tamion, A., Hillenkamp, M., Tournus, F., Bonet, E. & Dupuis, V. Accurate determination of the magnetic anisotropy in cluster-assembled nanostructures. *Appl. Phys. Lett.* **95**, 10–13 (2009).

4. El-Hilo, M. Nano-particle magnetism with a dispersion of particle sizes. *J. Appl. Phys.* **112**, (2012).

5. Laureti, S. *et al.* Magnetic interactions in silica coated nanoporous assemblies of CoFe 2O4 nanoparticles with cubic magnetic anisotropy. *Nanotechnology* **21**, (2010).

6. Hrubovčák, P., Zeleňáková, A., Zeleňák, V., Peddis, D. & Fiorani, D. Magnetic relaxation process determination in the Co/Au nanoparticle system. *Phys. Rev. B* **102**, 024433 (2020).
